# Global burden and cross-country inequalities of age-related eye diseases from 1990 to 2021: a comprehensive analysis of temporal trends and socioeconomic disparities

**DOI:** 10.1186/s40662-026-00473-5

**Published:** 2026-02-01

**Authors:** Kuan Li, Jing Tang, Zicheng Zhang, Xuyan Li, Yongxiang Zheng, Dan Jiang, Jie Sun

**Affiliations:** National Clinical Research Center for Ocular Diseases, Eye Hospital, Wenzhou Medical University, Wenzhou, 325027 China

**Keywords:** Age-related eye diseases, Age-related macular degeneration, Cataract, Glaucoma, Health inequalities, Global Burden of Disease, Temporal trends

## Abstract

**Background:**

Age-related eye diseases (AREDs) are leading causes of visual impairment worldwide. With global population aging, understanding their epidemiological trends and socioeconomic disparities is crucial for public health planning and equitable resource allocation.

**Methods:**

We conducted a secondary epidemiological analysis of AREDs using data from the Global Burden of Disease (GBD) Study 2021. We evaluated years lived with disability (YLDs) and age-standardized YLD rates (ASYR) and conducted trend analysis using Joinpoint regression. Cross-country inequalities were assessed using the slope index of inequality (SII) and concentration index, with correlation and regression analyses examining associations with the socio-demographic index (SDI).

**Results:**

Global YLDs for AREDs increased from 78.503 to 100.006, while ASYR decreased from 112.815 to 92.803 per 100,000 populations between 1990 and 2021. Despite a global increase in the relative burden of glaucoma, both absolute and relative inequalities for age-related macular degeneration (AMD) and cataracts decreased. Low-SDI countries showed slight improvements in reducing these inequalities. The SII for AREDs improved in lower-SDI countries between 1990 and 2021, with reductions in AMD (from − 9.250 to − 6.033), cataract (from − 258.131 to − 173.762), and glaucoma (from − 21.090 to − 20.064). The concentration index for AMD and cataract decreased from − 0.167 and − 0.335 in 1990 to − 0.129 and − 0.272 in 2021, respectively, while the concentration index for glaucoma increased from − 0.208 to − 0.263, Regional disparities in the AREDs burden were evident, with most regions showing improved inequality in lower-SDI countries as reflected in both the SII and concentration index.

**Conclusions:**

Despite global improvements in the relative burden of AREDs, significant socioeconomic and geographical inequalities persist, particularly in low-SDI regions. Targeted public health strategies and strengthened eye care systems are urgently needed to address these disparities and achieve equitable eye health outcomes worldwide.

**Supplementary Information:**

The online version contains supplementary material available at 10.1186/s40662-026-00473-5.

## Background

Age-related eye diseases (AREDs) are leading causes of visual impairment and blindness worldwide [1, 2]. AREDs encompass several pathologies, including age-related macular degeneration (AMD), cataract, glaucoma, diabetic retinopathy, age-related forms of uveitis and so on. Among these, AMD, cataract, and glaucoma are particularly notable for their direct and strong association with aging, and together they represent the most common causes of irreversible vision loss in the global elderly population [3]. As the global population continues to age, the burden imposed by these diseases is rising, making vision impairment an urgent public health priority [4]. According to the World Health Organization (WHO), over 1 billion people worldwide experienced vision impairment in 2019, with the majority of whom being individuals aged 50 and older [5, 6]. The Global Burden of Disease (GBD) Study has consistently shown that the prevalence of blindness and moderate-to-severe vision impairment has significantly increased from 1990 to 2020, reflecting both population aging and advances in diagnostic technology that have improved detection rates [7]. This trend is expected to continue as the global population ages, highlighting the growing challenge of managing vision impairment in aging populations.

The impact of AREDs extends beyond vision loss, as it is also closely associated with an elevated risk of social isolation, functional decline, and various medical comorbidities [8]. Severe visual impairment has been linked to higher rates of physical and cognitive impairment, dementia, and an increased risk of falls, particularly among the elderly [9]. Despite the significant socioeconomic burden posed by these diseases, early intervention and regular screening have been shown to reduce treatment costs and improve both individual outcomes and public health. Timely management reduces the economic healthcare costs and enhances the quality of life for affected individuals [10–12]. The GBD studies have been instrumental in tracking the landscape of disease burden over time. Previous studies have provided valuable insights into the prevalence of visual impairment in elderly populations across various regions [13, 14] and countries [15–17]. However, many existing studies are limited in scope, often focusing on single diseases, specific regions, or failing to systematically quantify cross-country socioeconomic inequalities. The recent release of the GBD 2021 dataset, with its extensive updates and incorporation of data up to 2021, offers an unprecedented opportunity for a more precise and contemporaneous assessment for AREDs burden.

In this study, we used the GBD 2021 data to conduct a comprehensive analysis of the global burden and cross-country inequalities of three major AREDs (AMD, cataract, and glaucoma) from 1990 to 2021. Our study aims to: (1) provide a detailed descriptive analysis of the temporal trends of these conditions at the global, regional, and national levels; (2) assess the association between their burden and the socio-demographic index (SDI) across regions and over time; and (3) quantify and compare cross-country inequalities, with particular focus on low-SDI regions.

## Methods

### Data source

We conducted a secondary analysis of the GBD 2021 data (https://vizhub.healthdata.org/gbd-results/). The GBD 2021 dataset provides an accessible epidemiological estimates for 371 diseases and injuries as well as 88 risk factors across 21 GBD regions and 204 countries/territories from 1990 to 2021, synthesizing data from nationally representative surveys, censuses, and meta-analyses [18–20]. ARED cases in the GBD database were identified through a comprehensive and standardized evidence synthesis framework that systematically integrates data from population-based surveys, national health information systems, hospital records, and published epidemiological studies [21, 22]. To ensure consistency and comparability, all data were harmonized using standardized case definitions for each ARED and adjusted for differences in study methodology, diagnostic criteria, and case ascertainment using Bayesian meta-regression models with DisMod-MR (version 2.1) [19, 23]. To address potential under-ascertainment and surveillance bias in low-income countries, the GBD methodology applies correction factors that account for incomplete surveillance, differential access to eye care, and non-random missing data [24]. These adjustments incorporate predictive covariates, such as healthcare access and quality indices, years of education, and other sociodemographic indicators, to model disease burden in regions with limited direct measurements [25, 26].

From this database, we extracted age, location, and gender-specific estimates of age-standardized years lived with disability rate (ASYR) along with their corresponding 95% uncertainty intervals (UIs) for AMD, cataract and glaucoma from 1990 to 2021. Data from 21 GBD regions and 204 countries were included. Additionally, we utilized the SDI as a composite measure of regional development based on the geometric mean of three factors: fertility rates under 25, education levels for adults over 15, and income per capita [27]. These factors are normalized on a scale from zero (indicating minimal development relevant to health) to one (indicating maximal development). This study adhered to the Standardized Reporting of Burden of Disease Studies (STROBOD) guidelines [28].

### Trend analysis

We calculated the standard errors based on the corresponding 95% UIs, obtained by dividing the width of the 95% UIs by 1.96 × 2. To evaluate temporal trends in the age-standardized rate over the 32-year study period and to identify specific years with the most significant changes, Joinpoint regression analysis [29] was used to calculate the annual percentage change (APC) and the average annual percentage change (AAPC). By allowing a maximum number of six joinpoints and using a Monte Carlo permutation method with 4499 randomly permuted data set, the Joinpoint regression analysis could find the best fit for joinpoints where significant changes in trend occurred. If both the APC/AAPC estimation and its lower boundary of the 95% confidence interval (CI) were both greater than 0, an increasing trend was identified for a given period. Conversely, if the APC/AAPC estimate and its upper boundary of the 95% CI were both lesser than 0, a decreasing trend was identified over a period of time. If the 95% CI overlapped zero, the trend was considered stable.

### Cross-country inequality analysis

The slope index of inequality (SII) and concentration index were calculated as standardized metrics to quantify inequalities in ASYR of AREDs globally and across the 21 GBD regions [30]. These metrics, recommended by WHO, measure absolute (SII) and relative (concentration index) inequality [31]. The SII was calculated through regression analysis, with ASYR at the country or territory level as the dependent variable. The midpoint of the cumulative class interval of the population, sorted by SDI, was used as the independent variable representing relative social position [32]. The concentration index was calculated using numerical integration under the Lorenz curve, which plots the cumulative proportion of ASYR against the cumulative proportion of the population, ranked by SDI [33, 34]. A positive SII/concentration index indicates that higher SDI is associated with a higher ASYR, and vice versa. Larger absolute values of the SII/concentration index indicate greater inequality.

### Statistical analysis

The ASYR was expressed as the estimate per 100,000 populations and 95% UIs. The SII, concentration index, and AAPC were all presented with their respective 95% CIs. Spearman's rank correlation analysis was used to assess the monotonic association between two continuous variables. Multivariable linear regression models, adjusted for relevant modifiable risk factors, were used to examine the associations between SDI and the ASYR of AREDs. A two‐sided *P* value of less than 0.05 was considered statistically significant. All statistical analyses and data visualizations were performed using R software (version 4.4.2).

## Results

### Global burden and temporal trends

**Fig. 1:**
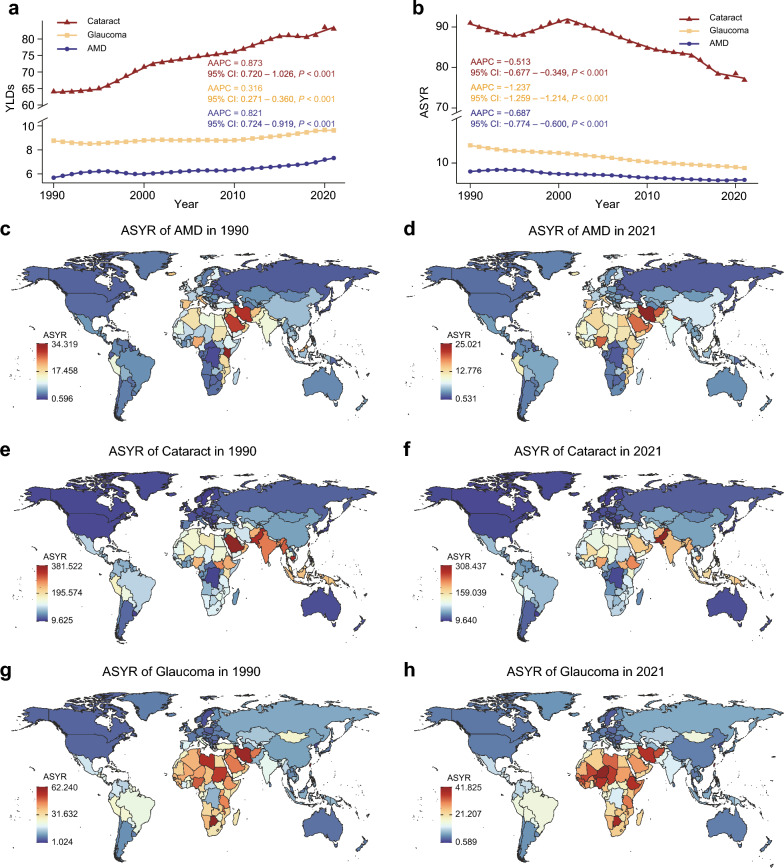
Global burden and temporal trends of age-related eye diseases. **a** Joinpoint regression analysis of years lived with disability (YLDs) for age-related eye diseases. **b** Joinpoint regression analysis of age-standardized years lived with disability rates (ASYRs) for age-related eye diseases. Global distribution of ASYR for age-related macular degeneration (AMD) (**c**, **d**), cataract (**e**, **f**), and glaucoma (**g**, **h**) in 1990 and 2021. AAPC, average annual percentage change; CI, confidence interval

Globally, the years lived with disability (YLDs) rate for AMD increased from 5.679 (95% UI: 3.871–7.911) per 100,000 populations in 1990 to 7.324 (95% UI: 5.085–10.107) in 2021, representing a 29% rise, with an AAPC of 0.821 (95% CI: 0.724–0.919, Table 1). In contrast, the ASYR for AMD declined from 8.382 (95% UI: 5.697–11.530) to 6.782 (95% UI: 4.704–9.317) per 100,000 populations during the same period, with an AAPC of − 0.687 (95% CI: − 0.774 to − 0.600) (Table 1). In 2021, Western Sub-Saharan Africa showed the highest ASYR (14.735; 95% UI: 10.431–20.564), while the Caribbean had the lowest (1.777; 95% UI: 1.170–2.503) per 100,000 populations. Between 1990 and 2021, all GBD regions showed negative AAPC values for the ASYR of AMD, except for Central and Southern Sub-Saharan Africa (Table 2). The top five countries with the highest ASYR for AMD in 2021 were Iran, Nepal, Afghanistan, Nigeria, and Saudi Arabia (Fig. 1d, Table S3). Sex-specific analyses indicated consistently higher ASYR among females compared to males throughout the study period, although both groups showed declining trends (Table 2).

**Table 1:** Global trends in ASYR and YLD for three major age-related eye diseases from 1990 to 2021

Disease	ASYR per 100,000		YLDs per 100,000	
	AAPC (95% CI)	*P* value	AAPC (95% CI)	*P* value
Age-related macular degeneration	− 0.687 (− 0.774 to − 0.600)	< 0.001	0.821 (0.724–0.919)	< 0.001
Cataract	− 0.516 (− 0.688 to − 0.343)	< 0.001	0.873 (0.720–1.026)	< 0.001
Glaucoma	− 1.237 (− 1.264 to − 1.211)	< 0.001	0.316 (0.271–0.360)	< 0.001

*ASYR* = age-standardized of years lived with disability rate; *YLDs* = years lived with disability; *AAPC* = average annual percent change; *CI* = confidence interval

**Table 2:** ASYRs and AAPC of AREDs by sex, region, and socio-demographic index from 1990 to 2021

	ASYR of AMD		ASYR of cataract		ASYR of glaucoma	
	AAPC (95% CI)	*P* value	AAPC (95% CI)	*P* value	AAPC (95% CI)	*P* value
Global	− 0.687 (− 0.774 to − 0.600)	< 0.001	− 0.516 (− 0.688 to − 0.343)	< 0.001	− 1.237 (− 1.264 to − 1.211)	< 0.001
Sex						
Male	− 0.652 (− 0.750 to − 0.554)	< 0.001	− 0.751 (− 0.891 to − 0.612)	< 0.001	− 1.402 (− 1.429 to − 1.374)	< 0.001
Female	− 0.651 (− 0.706 to − 0.595)	< 0.001	− 0.353 (− 0.550 to − 0.157)	< 0.001	− 1.121 (− 1.143 to − 1.098)	< 0.001
Region						
Andean Latin America	− 0.510 (− 0.546 to − 0.473)	< 0.001	− 1.421 (− 1.470 to − 1.371)	< 0.001	− 1.594 (− 1.657 to − 1.531)	< 0.001
Australasia	− 0.830 (− 0.891 to − 0.768)	< 0.001	− 0.304 (− 0.339 to − 0.269)	< 0.001	− 0.767 (− 0.814 to − 0.721)	< 0.001
Caribbean	− 0.675 (− 0.730 to − 0.619)	< 0.001	− 1.051 (− 1.072 to − 1.030)	< 0.001	− 1.328 (− 1.351 to − 1.306)	< 0.001
Central Asia	− 0.237 (− 0.307 to − 0.167)	< 0.001	− 0.545 (− 0.562 to − 0.528)	< 0.001	− 0.991 (− 1.012 to − 0.970)	< 0.001
Central Europe	− 0.534 (− 0.690 to − 0.378)	< 0.001	− 0.414 (− 0.424 to − 0.404)	< 0.001	− 1.189 (− 1.226 to − 1.151)	< 0.001
Central Latin America	− 0.828 (− 0.851 to − 0.805)	< 0.001	− 1.226 (− 1.267 to − 1.184)	< 0.001	− 1.465 (− 1.490 to − 1.440)	< 0.001
Central Sub-Saharan Africa	0.047 (0.000 to 0.095)	0.050	− 0.727 (− 0.744 to − 0.709)	< 0.001	− 0.553 (− 0.582 to − 0.523)	< 0.001
East Asia	− 0.078 (− 0.201 to 0.045)	0.214	− 0.436 (− 0.697 to − 0.176)	0.001	− 2.445 (− 2.621 to − 2.270)	< 0.001
Eastern Europe	− 0.627 (− 0.667 to − 0.587)	< 0.001	− 0.580 (− 0.613 to − 0.547)	< 0.001	− 1.115 (− 1.144 to − 1.086)	< 0.001
Eastern Sub-Saharan Africa	− 0.860 (− 0.968 to − 0.751)	< 0.001	− 0.604 (− 0.640 to − 0.568)	< 0.001	− 0.895 (− 0.932 to − 0.858)	< 0.001
High-income Asia Pacific	− 0.748 (− 0.787 to − 0.708)	< 0.001	− 0.409 (− 0.449 to − 0.369)	< 0.001	− 0.923 (− 0.965 to − 0.880)	< 0.001
High-income North America	− 0.492 (− 0.561 to − 0.423)	< 0.001	− 0.199 (− 0.257 to − 0.140)	< 0.001	− 0.336 (− 0.405 to − 0.266)	< 0.001
North Africa and Middle East	− 0.554 (− 0.608 to − 0.501)	< 0.001	− 1.178 (− 1.213 to − 1.142)	< 0.001	− 1.545 (− 1.662 to − 1.428)	< 0.001
Oceania	− 0.825 (− 0.921 to − 0.728)	< 0.001	− 0.597 (− 0.620 to − 0.574)	< 0.001	− 0.854 (− 0.980 to − 0.728)	< 0.001
South Asia	− 1.496 (− 1.569 to − 1.424)	< 0.001	− 1.211 (− 1.290 to − 1.131)	< 0.001	− 1.525 (− 1.766 to − 1.283)	< 0.001
Southeast Asia	− 1.212 (− 1.268 to − 1.156)	< 0.001	− 1.210 (− 1.241 to − 1.179)	< 0.001	− 1.574 (− 1.622 to − 1.527)	< 0.001
Southern Latin America	− 0.938 (− 0.979 to − 0.897)	< 0.001	− 0.711 (− 0.724 to − 0.698)	< 0.001	− 1.211 (− 1.254 to − 1.167)	< 0.001
Southern Sub-Saharan Africa	0.086 (0.031 to 0.140)	0.002	− 1.132 (− 1.177 to − 1.088)	< 0.001	− 0.742 (− 0.900 to − 0.584)	< 0.001
Tropical Latin America	− 0.275 (− 0.363 to − 0.187)	< 0.001	− 1.199 (− 1.285 to − 1.113)	< 0.001	− 1.107 (− 1.160 to − 1.054)	< 0.001
Western Europe	− 1.048 (− 1.079 to − 1.017)	< 0.001	− 0.309 (− 0.332 to − 0.286)	< 0.001	− 1.175 (− 1.204 to − 1.147)	< 0.001
Western Sub-Saharan Africa	− 0.176 (− 0.270 to − 0.083)	< 0.001	− 0.411 (− 0.436 to − 0.386)	< 0.001	− 0.730 (− 0.818 to − 0.643)	< 0.001
SDI region						
Low	− 0.533 (− 0.593 to − 0.474)	< 0.001	− 0.787 (− 0.823 to − 0.751)	< 0.001	− 1.015 (− 1.092 to − 0.937)	< 0.001
Low–Middle	− 1.289 (− 1.338 to − 1.240)	< 0.001	− 1.146 (− 1.207 to − 1.084)	< 0.001	− 1.503 (− 1.643 to − 1.362)	< 0.001
Middle	− 0.558 (− 0.709 to − 0.407)	< 0.001	− 0.847 (− 1.064 to − 0.629)	< 0.001	− 1.600 (− 1.672 to − 1.528)	< 0.001
High–Middle	− 0.499 (− 0.637 to − 0.361)	< 0.001	− 0.101 (− 0.254 to 0.052)	0.194	− 1.700 (− 1.819 to − 1.582)	< 0.001
High	− 0.952 (− 0.979 to − 0.924)	< 0.001	− 0.238 (− 0.297 to − 0.178)	< 0.001	− 0.836 (− 0.864 to − 0.809)	< 0.001

*ASYR* = age-standardized of years lived with disability rate (per 100,000); *AAPC* = average annual percent change; *SDI* = socio-demographic index; *CI* = confidence interval

For cataract burden, the global YLDs rate for cataract increased from 64.057 (95% UI: 46.406–85.219) to 83.051 (95% UI: 60.016–111.569) per 100,000 populations, with an AAPC of 0.873 (95% CI: 0.720–1.026) from 1990 to 2021 (Table 1). During the same period, the ASYR decreased from 91.062 (95% UI: 66.260–120.396) to 76.967 (95% UI: 55.638–103.498) per 100,000, with an AAPC of − 0.516 (95% UI: − 0.688 to − 0.343) (Table 1). In 2021, South Asia exhibited the highest regional ASYR (193.717; 95% UI: 141.641–256.307) per 100,000 populations, whereas high-income North America had the lowest ASYR (16.611; 95% UI: 11.774–22.295). All GBD regions showed negative AAPCs for cataract ASYR from 1990 to 2021 (Table 2). Thailand, Myanmar, Cook Islands, India, and Pakistan had the highest national ASYR values in 2021 (Fig. 1f, Table S4). Females consistently showed higher ASYR for cataract than males, though both sexes showed declining trends over time (Table 2).

Between 1990 and 2021, the global YLDs rate for glaucoma increased from 8.767 (95% UI: 6.065–12.161) to 9.630 (95% UI: 6.728–13.295) per 100,000, with an AAPC of 0.316 (95% CI: 0.271–0.360) (Table 1). Meanwhile, the ASYR for glaucoma decreased from 13.371 (95% UI: 9.358–18.497) to 9.054 (95% UI: 6.292–12.456), with an AAPC of − 1.237 (95% UI: − 1.264 to − 1.211) (Table 1). Western Sub-Saharan Africa showed the highest regional ASYR for glaucoma (32.481; 95% UI: 22.354 to 44.526), and Central Europe the lowest (3.539; 95% UI: 2.433–4.866) per 100,000 in 2021. All GBD regions exhibited negative AAPCs for glaucoma ASYR from 1990 to 2021 (Table 2). Niger, Botswana, Ethiopia, Iran, and Nigeria were the five countries with the highest glaucoma ASYR in 2021 (Fig. 1h, Table S5). Males showed higher ASYR values than females across the study period, although both groups demonstrated decreasing trends (Table 2).

### Relationship between AREDs burdens and SDI

**Fig. 2:**
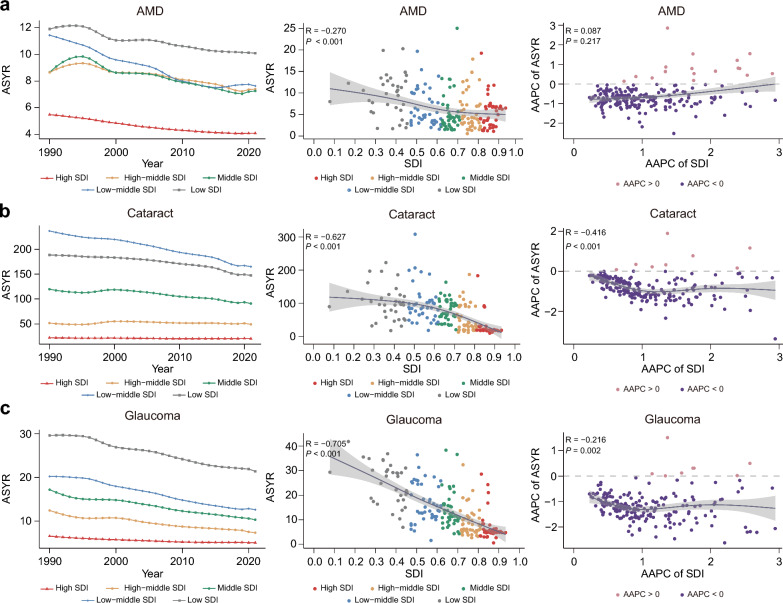
Association between socio-demographic index (SDI) and age-standardized years lived with disability rates (ASYRs) of age-related eye diseases. Left panels: temporal trends of ASYR across SDI quintiles, 1990–2021. Middle panels: correlation between SDI and ASYR in 2021. Right panels: association between average annual percentage changes (AAPC) of SDI and ASYR, 1990–2021. **a** Age-related macular degeneration (AMD); **b** Cataract; **c** Glaucoma

In 2021, the low-SDI region exhibited the highest ASYR for AMD (10.082; 95% UI: 6.906–13.860). Between 1990 and 2021, the low-middle SDI region showed the most pronounced decline in ASYR, with an AAPC of − 1.289 (95% UI: − 1.338 to − 1.240) (Table 2). Correlation analysis of 2021 data indicated a weak negative correlation between SDI and ASYR for AMD in 2021 (R =  − 0.270, *P* < 0.001). Multivariable regression analysis confirmed that higher SDI was associated with a lower ASYR of AMD (β =  − 0.350, SE = 0.037, 95% CI: − 0.423 to − 0.277, t =  − 9.571, *P* < 0.001) (Table S6). However, no significant linear relationship was observed between the AAPC of SDI and the AAPC of AMD ASYR from 1990 to 2021 (R = 0.087, *P* = 0.217) (Fig. 2a).

For cataract and SDI*,* the low-middle SDI region exhibited the highest ASYR for cataract (164.864; 95% UI: 119.714–218.194) in 2021, followed by the low-SDI region (147.260; 95% UI: 106.885–195.014). Between 1990 and 2021, the low-middle SDI region showed the highest negative AAPC in ASYR for cataract at − 1.146 (95% UI: − 1.207 to − 1.084) (Table 2). A strong negative correlation was observed between SDI and ASYR for cataract in 2021 (R =  − 0.627, *P* < 0.001). Multivariable regression further supported this relationship (β =  − 0.134, SE = 0.037, 95% CI: − 0.207 to − 0.061, t =  − 3.600, *P* < 0.001) (Table S6). Additionally, a moderate negative correlation was found between the annual changes in SDI and in cataract ASYR over time (R =  − 0.416, *P* < 0.001) (Fig. 2b).

For glaucoma and SDI, the highest glaucoma ASYR was observed in the Low SDI region (21.388; 95% UI: 14.631–29.415), followed by the low-middle SDI region (12.615; 95% UI: 8.652–17.138). The high-middle SDI region showed the most rapid decline in ASYR from 1990 to 2021 (AAPC =  − 1.700; 95% CI: − 1.819 to − 1.582) (Table 2). A strong negative correlation was identified between SDI and glaucoma ASYR in 2021 (R =  − 0.705, *P* < 0.001). Multivariable analysis confirmed that SDI was negatively associated with glaucoma ASYR (β =  − 0.213, SE = 0.029, 95% CI: − 0.270 to − 0.156, t =  − 7.112,* P* < 0.001) (Table S6). A weak negative correlation was also observed between the annual changes in SDI and in glaucoma ASYR (R =  − 0.216, *P* = 0.002) (Fig. 2c).

### Cross-country inequalities of AREDs

From 1990 to 2021, the SII and concentration index for all three AREDs were all negative globally (Figure S1 and Figure S2), indicating inequality in the distribution of disease burden. Considerable heterogeneity in inequality patterns was observed across GBD regions (Figure S1 and Figure S2).

**Fig. 3:**
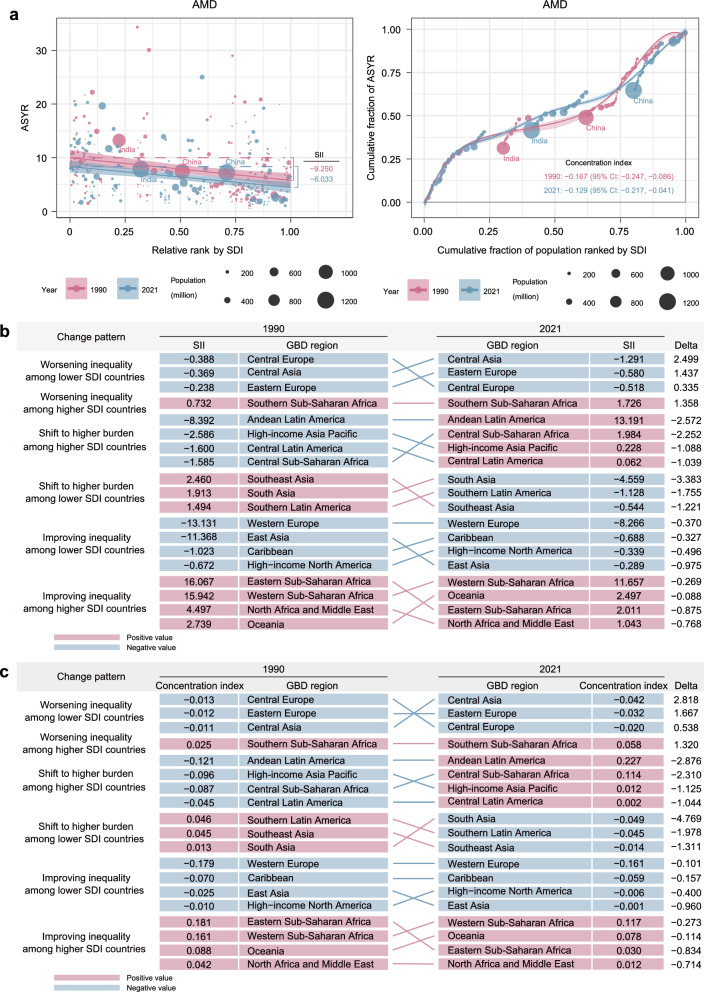
Cross-country inequalities in age-related macular degeneration (AMD) burden. **a** Absolute inequalities (slope index of inequality [SII]) and relative inequalities (concentration index) at global level. **b** Regional patterns of absolute inequality changes. **c** Regional patterns of relative inequality changes. ASYR, age-standardized years lived with disability rate; GBD, Global Burden of Disease; SDI, socio-demographic index

The global AMD ASYR burden was primarily concentrated in lower-SDI countries in both 1990 and 2021(Fig. 3a). From 1990 to 2021, the SII for AMD showed an improvement in absolute inequality, with values decreasing from − 9.250 (95% CI: − 11.590 to − 6.909) to − 6.033 (95% CI: − 7.976 to − 4.090) (Table S7). Similarly, the concentration index improved from − 0.167 (95% CI: − 0.247 to − 0.086) to − 0.129 (95% CI: − 0.217 to − 0.041), reflecting a reduction in relative inequality (Table S8, Fig. 3b). Substantial regional heterogeneity in AMD inequality changes was observed between 1990 and 2021. The pattern of inequality shifts was roughly uniformly distributed across regions, with Central Europe showing the largest worsening inequality among lower-SDI countries in both the SII and concentration index. Southern Sub-Saharan Africa was the only region reporting worsening inequality among higher-SDI countries. In 2021, Western Europe recorded both the highest negative SII of − 8.266 (95% CI: − 11.460 to − 5.072) and concentration index of − 0.161 (95% CI: − 0.267 to − 0.054), while East Asia had the lowest negative SII of − 0.289 (95% CI: − 22.704 to 22.126) and concentration index of − 0.001 (95% CI: − 0.112 to 0.111) (Table S9, Table S10).

**Fig. 4:**
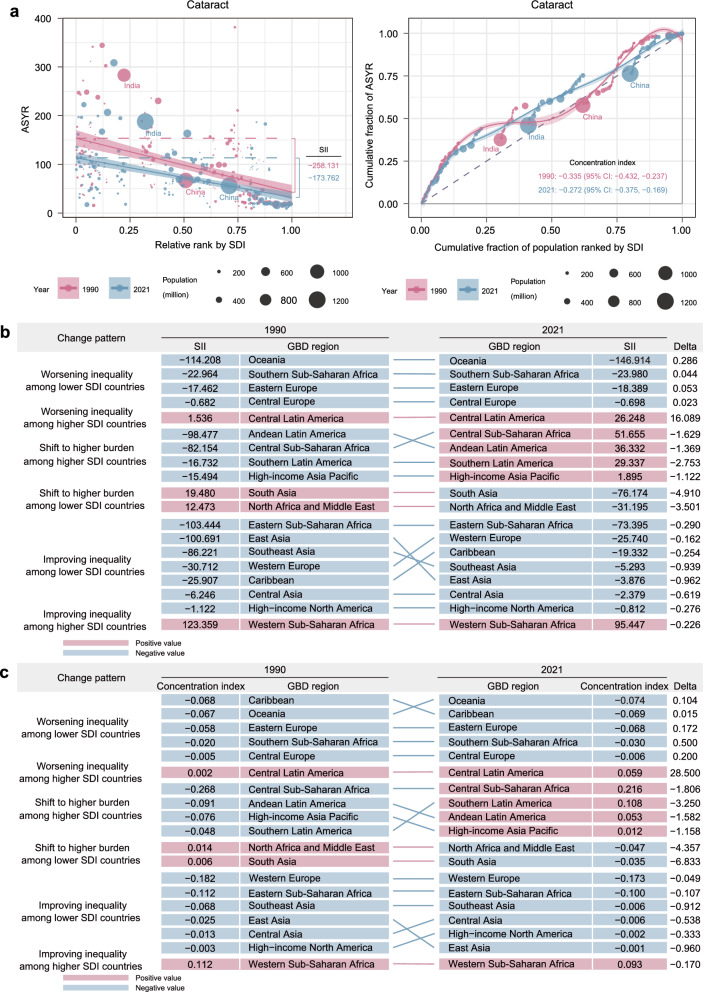
Cross-country inequalities in cataract burden. **a** Absolute inequalities (slope index of inequality [SII]) and relative inequalities (concentration index) at global level. **b** Regional patterns of absolute inequality changes. **c** Regional patterns of relative inequality changes. ASYR, age-standardized years lived with disability rate; GBD, Global Burden of Disease; SDI, socio-demographic index

The ASYR burden for cataract predominantly affected lower-SDI countries in both 1990 and 2021 (Fig. 4a). From 1990 to 2021, the SII for cataract decreased from − 258.131 (95% CI: − 291.377 to − 224.885) to − 173.762 (95% CI: − 199.899 to − 147.624), indicating a reduction in absolute inequality (Table S7). The concentration index for cataract ASYR also improved from − 0.335 (95% CI: − 0.432 to − 0.237) to − 0.272 (95% CI: − 0.375 to − 0.169), indicating reduced relative inequality in lower-SDI countries (Table S8, Fig. 4b).

Most regions showed improving inequality in lower-SDI countries, with East Asia exhibiting greatest improvement in both the SII and concentration index. Oceania exhibited the largest worsening inequality in SII among lower-SDI countries, showing the highest negative SII of − 146.914 (95% CI: − 167.425 to − 126.402) in 2021, while Central Europe had the lowest negative SII of − 0.698 (95% CI: − 7.896 to 6.499, Table S9). Western Europe recorded the highest negative concentration index of − 0.173 (95% CI: − 0.283 to − 0.064), and East Asia showed the lowest negative concentration index of − 0.001 (95% CI: − 0.114 to 0.112, Table S10).

**Fig. 5:**
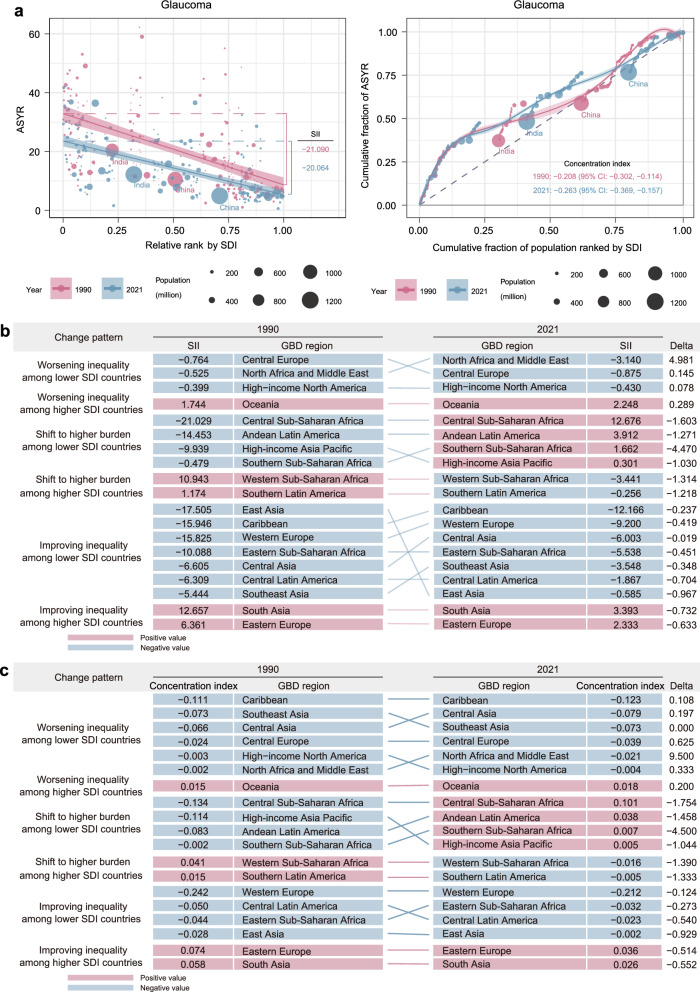
Cross-country inequalities in glaucoma burden. **a** Absolute inequalities (slope index of inequality [SII]) and relative inequalities (concentration index) at global level. **b** Regional patterns of absolute inequality changes. **c** Regional patterns of relative inequality changes. ASYR, age-standardized years lived with disability rate; GBD, Global Burden of Disease; SDI, socio-demographic index

The ASYR burden of glaucoma globally was predominantly concentrated in lower-SDI countries in both 1990 and 2021 (Fig. 5a). Between 1990 and 2021, the SII for glaucoma showed an improvement in absolute inequality, with the value decreasing from − 21.090 (95% CI: − 25.597 to − 16.583) to − 20.064 (95% CI: − 23.475 to − 16.653) (Table S7). However, the concentration index for glaucoma ASYR worsened in lower-SDI countries, increasing from − 0.208 (95% CI: − 0.302 to − 0.114) to − 0.263 (95% CI: − 0.369 to − 0.157), indicating an increase in relative inequality (Table S8, Fig. 5b). Regionally, most regions reported an improving inequality among lower-SDI countries in SII, with East Asia showing the largest improvement. In contrast, most regions exhibited worsening relative inequality (concentration index), with North Africa and Middle East showing the largest increases in inequality. In 2021, the Caribbean recorded the highest negative SII of − 12.166 (95% CI: − 14.543 to − 9.789) and the highest negative concentration index of − 0.123 (95% CI: − 0.194 to − 0.052). Southern Latin America reported the lowest negative SII of − 0.256 (95% CI: − 1.239 to 0.727) while East Asia showed the lowest negative concentration index of − 0.002 (95% CI: − 0.173 to 0.169) (Table S9, Table S10).

## Discussion

Our study provides a comprehensive analysis of the global burden and socioeconomic inequalities in three major AREDs from 1990 to 2021, revealing both significant progress and persistent challenges in global eye health. Our systematic analysis demonstrates divergent trends: while absolute burden measured by YLDs has increased, ASYR has declined across all three AREDs, reflecting the complex interplay between demographic aging and public health interventions.

The observed increase in YLDs for AMD, cataract, and glaucoma underscores the growing impact of global population aging on eye health systems worldwide [35–37]. In contrast, the decline in ASYR suggests progress in eye care management and possibly broader public health improvements at the global level and more effective therapies, such as anti-vascular endothelial growth factor for neovascular AMD [38–40], improvements in cataract surgical coverage and outcomes [41, 42], and advancement in early detection and management strategies for AREDs using artificial intelligence (AI) and teleophthalmology [43–53]. However, the strong negative correlations we observed between SDI and the ASYR of cataract and glaucoma highlight that this progress has been profoundly uneven. The burden remains disproportionately concentrated in lower-SDI countries. This association is less pronounced for AMD, a finding that may reflect fundamental differences in disease pathophysiology and treatment availability. While cataract is largely treatable with surgical intervention [54, 55] and glaucoma management relies heavily on continuous monitoring and access to care [56], the majority of AMD cases are of the dry subtype, for which effective disease-modifying therapies remain limited [57, 58]. Consequently, the AMD burden may be less sensitive to variations in healthcare system capacity captured by the SDI. The substantial burden of cataract in low-SDI regions is driven by a confluence of modifiable risk factors including smoking, household air pollution from solid fuels, and increasingly, ambient particulate matter (PM2.5) exposure [59, 60], and its impact extends far beyond vision loss, having profound implications for overall health and quality of life in older adults, being associated with increased risks of falls, functional decline, and notably, dementia. This underscores that interventions for cataract are not merely vision-saving but are integral to healthy aging. The concentration of these risk factors and the severe downstream consequences in low-SDI settings highlight an urgent need for integrated public health strategies that combine eye care with broader environmental and aging-health initiatives. Our study further highlights significant gender disparities in the burden of AREDs, with females generally showing higher ASYR values for AREDs. These observed differences are likely influenced by demographic, biological, and socioeconomic factors. Firstly, women generally have a longer life expectancy than men, leading to a larger population of elderly females who are at high risk for AREDs. Secondly, hormonal differences may contribute to this disparity. Emerging evidence suggests that post-menopausal estrogen depletion has been implicated in the pathogenesis of AREDs [61]. Furthermore, socioeconomic inequities in healthcare access significantly impact these trends. Despite the global reduction in the burden of AREDs, women in many low- and middle-income regions continue to face greater barriers to accessing cataract surgery and other eye care services compared to men [62].

Compared to simpler comparative measures, such as the disability-adjusted life year (DALY) rate ratio between the highest- and lowest-ranking countries, the SII and concentration index used in this study offers significant advantages, including the comprehensive use of the entire SDI distribution rather than focusing solely on the extremes, formal statistical inference which supports comparability over time and across regions and complementary measures of SII (as an absolute measure) and the concentration index (as a relative measure) which can help avoid the potential paradox where absolute and relative gaps move in opposite directions as overall rates change [27]. Inequality analyses showed that the ASYR burden for AREDs as predominantly concentrated in lower-SDI countries in both 1990 and 2021, reflecting the disproportionate burden faced by less developed regions. Our findings also underscore the disproportionate prevalence of AREDs in Sub-Saharan Africa and Oceania, largely due to regional inequities in healthcare access and socioeconomic factors. In Sub-Saharan Africa, limited healthcare infrastructure and high levels of poverty hinder early diagnosis and treatment, leading to higher rates of blindness [63]. Similarly, in Oceania, geographic isolation and inadequate service provision exacerbate the burden of eye disease, leading to more severe eye health issues in these communities [64]. Significant regional variations in inequality patterns were observed between 1990 and 2021. East Asia recorded the lowest negative concentration index for all three diseases in 2021, reflecting the successful implementation of eye care policies and interventions. On the other hand, Western Europe, despite its high healthcare standards, continued to exhibit significant socioeconomic inequalities in the burden of AMD and cataracts.

Several limitations should be considered when interpreting our findings. First, AREDs cases in developing countries reported in the GBD might be underestimated due to systemic infrastructure issues, including limited surveillance systems and insufficient healthcare workforce relative to population demands. Such conditions may lead to underdiagnosis and lost documentation, among other problems [65, 66]. Second, healthcare resource indicators such as ophthalmologist density and diagnostic capacity are not available in the publicly accessible GBD dataset which limited our ability to fully account for health system disparities across countries. Future studies should incorporate standardized healthcare resource metrics when such data becomes available. Lastly, as a secondary analysis of GBD 2021 data, we were constrained by the available country-year-sex time-series estimates, which precluded full integration of other vision-threatening conditions like uveitis and diabetic retinopathy into our primary analytical framework.

## Conclusions

This study underscores the importance of addressing socioeconomic disparities in the burden of AREDs through targeted public health interventions. We demonstrated that while substantial progress were made in reducing the relative burden of AREDs globally, profound inequalities persist along socioeconomic, geographic, and gender dimensions. Reducing inequities will require strengthening health systems, promoting early diagnosis, and prioritizing interventions in high-burden regions. Future efforts should focus on integrating standardized healthcare resource metrics into burden analyses, developing and scaling up cost-effective technological solutions like portable devices and AI, and implementing targeted, gender-sensitive, and community-engaged eye care programs.

## Supplementary Information

Supplementary material 1.

## Data Availability

The datasets analyzed in this study are available in the GBD 2021 database (https://gbd2021.healthdata.org/gbd-results/) and the SDI information was downloaded from https://ghdx.healthdata.org/record/global-burden-disease-study-2021-gbd-2021-socio-demographic-index-sdi-1950%E2%80%932021. All analytical code used for data processing, statistical analysis, and visualization in this secondary analysis are deposited in a publicly accessible GitHub repository (https://github.com/ugleek/GBD-AREDs).

## Abbreviations

AAPC: Average annual percent change
AI: Artificial intelligence
AMD: Age-related macular degeneration
APC: Annual percentage change
AREDs: Age-related eye diseases
ASYR: Age-standardized years lived with disability rate
CI: Confidence interval
DALYs: Disability-adjusted life years
GBD: Global Burden of Disease
SDI: Socio-demographic index
SII: Slope index of inequality
STROBOD: Standardized Reporting of Burden of Disease Studies
UI: Uncertainty interval
WHO: World Health Organization
YLDs: Years lived with disability

## References

[1] Evans JR, Fletcher AE, Wormald RP. Causes of visual impairment in people aged 75 years and older in Britain: an add-on study to the MRC Trial of Assessment and Management of Older People in the Community. Br J Ophthalmol. 2004;88(3):365–70. 14977771 10.1136/bjo.2003.019927 PMC1772038

[2] Eichenbaum JW. Geriatric vision loss due to cataracts, macular degeneration, and glaucoma. Mt Sinai J Med. 2012;79(2):276–94. 22499498 10.1002/msj.21303

[3] You L, Lin Y, Zheng Y, Han Z, Zeng L, Chen H. The impact of aging on ocular diseases: unveiling complex interactions. Aging Dis. 2024;16(5):2803–30. 39500360 10.14336/AD.2024.0850 PMC12339180

[4] Klein R, Klein BE. The prevalence of age-related eye diseases and visual impairment in aging: current estimates. Invest Ophthalmol Vis Sci. 2013;54(14):ORSF5–13. 24335069 10.1167/iovs.13-12789 PMC4139275

[5] World Health Organization. World report on vision**.** https://www.who.int/publications/i/item/9789241516570. Accessed June 2025.

[6] Think Global Health. Putting Vision Loss and Aging into Focus. https://www.thinkglobalhealth.org/article/putting-vision-loss-and-aging-focus. Accessed June 2025.

[7] GBD 2019 Blindness and vision impairment collaborators, vision loss expert group of the global burden of disease study. Trends in prevalence of blindness and distance and near vision impairment over 30 years: an analysis for the Global Burden of Disease Study. Lancet Glob Health. 2021;9(2):e130–43.

[8] Jacobs JM, Hammerman-Rozenberg R, Maaravi Y, Cohen A, Stessman J. The impact of visual impairment on health, function and mortality. Aging Clin Exp Res. 2005;17(4):281–6. 16285193 10.1007/BF03324611

[9] Hajek A, Brettschneider C, Lühmann D, Eisele M, Mamone S, Wiese B, et al. Effect of visual impairment on physical and cognitive function in old age: findings of a population-based prospective cohort study in Germany. J Am Geriatr Soc. 2016;64(11):2311–6. 27676141 10.1111/jgs.14458

[10] Brown GC, Brown MM, Busbee BG. Cost-utility analysis of cataract surgery in the United States for the year 2018. J Cataract Refract Surg. 2019;45(7):927–38. 31262482 10.1016/j.jcrs.2019.02.006

[11] Hopley C, Salkeld G, Wang JJ, Mitchell P. Cost utility of screening and treatment for early age related macular degeneration with zinc and antioxidants. Br J Ophthalmol. 2004;88(4):450–4. 15031152 10.1136/bjo.2003.035279 PMC1772079

[12] Peeters A, Schouten JS, Webers CA, Prins MH, Hendrikse F, Severens JL. Cost-effectiveness of early detection and treatment of ocular hypertension and primary open-angle glaucoma by the ophthalmologist. Eye (Lond). 2008;22(3):354–62. 17128205 10.1038/sj.eye.6702637

[13] Vision Loss Expert Group of The Global Burden of Disease Study, The Gbd Blindness and Vision Impairment Collaborators. Prevalence of vision loss in Latin America and the Caribbean in 2020: magnitude and temporal trends. Ophthalmic Epidemiol. 2025;33(1):36–42. 40418103 10.1080/09286586.2025.2464168

[14] Hsu WM, Cheng CY, Liu JH, Tsai SY, Chou P. Prevalence and causes of visual impairment in an elderly Chinese population in Taiwan: the Shihpai Eye Study. Ophthalmology. 2004;111(1):62–9. 14711715 10.1016/j.ophtha.2003.05.011

[15] Killeen OJ, De Lott LB, Zhou Y, Hu M, Rein D, Reed N, et al. Population prevalence of vision impairment in US adults 71 years and older: The National Health and Aging Trends Study. JAMA Ophthalmol. 2023;141(2):197–204. 36633858 10.1001/jamaophthalmol.2022.5840 PMC9857701

[16] Ehrlich JR, Agarwal A, Young C, Lee J, Bloom DE. The prevalence of vision impairment and blindness among older adults in India: findings from the Longitudinal Ageing Study in India. Nat Aging. 2022;2(11):1000–7. 37118083 10.1038/s43587-022-00298-6 PMC10148950

[17] Yin J, Jiang B, Zhao T, Guo X, Tan Y, Wang Y. Trends in the global burden of vision loss among the older adults from 1990 to 2019. Front Public Health. 2024;12:1324141. 38638474 10.3389/fpubh.2024.1324141 PMC11025641

[18] Vision Loss Expert Group of the Global Burden of Disease Study, GBD 2019 Blindness and Vision Impairment Collaborators. Global estimates on the number of people blind or visually impaired by glaucoma: a meta-analysis from 2000 to 2020. Eye (Lond). 2024;38(11):2036–46. 38565601 10.1038/s41433-024-02995-5 PMC11269708

[19] GBD 2021 Diseases and Injuries Collaborators. Global incidence, prevalence, years lived with disability (YLDs), disability-adjusted life-years (DALYs), and healthy life expectancy (HALE) for 371 diseases and injuries in 204 countries and territories and 811 subnational locations, 1990-2021: a systematic analysis for the Global Burden of Disease Study 2021. Lancet. 2024;403(10440):2133–61. 38642570 10.1016/S0140-6736(24)00757-8 PMC11122111

[20] GBD 2021 Risk Factors Collaborators. Global burden and strength of evidence for 88 risk factors in 204 countries and 811 subnational locations, 1990–2021: a systematic analysis for the Global Burden of Disease Study 2021. Lancet. 2024;403(10440):2162–203. 38762324 10.1016/S0140-6736(24)00933-4 PMC11120204

[21] GBD 2019 Blindness and Vision Impairment Collaborators, Vision Loss Expert Group of the Global Burden of Disease Study. Causes of blindness and vision impairment in 2020 and trends over 30 years, and prevalence of avoidable blindness in relation to VISION 2020: the right to sight: an analysis for the Global Burden of Disease Study. Lancet Glob Health. 2021;9(2):e144–60. 33275949 10.1016/S2214-109X(20)30489-7 PMC7820391

[22] GBD 2019 Diseases and Injuries Collaborators. Global burden of 369 diseases and injuries in 204 countries and territories, 1990–2019: a systematic analysis for the Global Burden of Disease Study 2019. Lancet. 2020;396(10258):1204–22. 33069326 10.1016/S0140-6736(20)30925-9 PMC7567026

[23] GBD 2017 Disease and Injury Incidence and Prevalence Collaborators. Global, regional, and national incidence, prevalence, and years lived with disability for 354 diseases and injuries for 195 countries and territories, 1990–2017: a systematic analysis for the Global Burden of Disease Study 2017. Lancet. 2018;392(10159):1789–858. 30496104 10.1016/S0140-6736(18)32279-7 PMC6227754

[24] GBD 2019 Ageing Collaborators. Global, regional, and national burden of diseases and injuries for adults 70 years and older: systematic analysis for the Global Burden of Disease 2019 Study. BMJ. 2022;376:e068208. 35273014 10.1136/bmj-2021-068208 PMC9316948

[25] GBD 2016 Healthcare Access and Quality Collaborators. Measuring performance on the Healthcare Access and Quality Index for 195 countries and territories and selected subnational locations: a systematic analysis from the Global Burden of Disease Study 2016. Lancet. 2018;391(10136):2236–71. 29893224 10.1016/S0140-6736(18)30994-2 PMC5986687

[26] GBD 2015 Disease and Injury Incidence and Prevalence Collaborators. Global, regional, and national incidence, prevalence, and years lived with disability for 310 diseases and injuries, 1990–2015: a systematic analysis for the Global Burden of Disease Study 2015. Lancet. 2016;388(10053):1545–602. 27733282 10.1016/S0140-6736(16)31678-6 PMC5055577

[27] Schlotheuber A, Hosseinpoor AR. Summary measures of health inequality: a review of existing measures and their application. Int J Environ Res Public Health. 2022;19(6):3697. 35329383 10.3390/ijerph19063697 PMC8992138

[28] Devleesschauwer B, Charalampous P, Gorasso V, Assunção R, Hilderink H, Idavain J, et al. Standardised reporting of burden of disease studies: the STROBOD statement. Popul Health Metr. 2024;22(1):28. 39375690 10.1186/s12963-024-00347-9 PMC11459887

[29] National Cancer Institute. Joinpoint Trend Analysis Software (Version 5.3.0). https://surveillance.cancer.gov/joinpoint/. Accessed Nov 2024.

[30] Luo Z, Shan S, Cao J, Zhou J, Zhou L, Jiang D, et al. Temporal trends in cross-country inequalities of stroke and subtypes burden from 1990 to 2021: a secondary analysis of the global burden of disease study 2021. EClin Med. 2024;76:102829. 10.1016/j.eclinm.2024.102829 PMC11415963 39309727

[31] Hosseinpoor AR, Bergen N, Schlotheuber A. Promoting health equity: WHO health inequality monitoring at global and national levels. Glob Health Action. 2015;8:29034. 26387506 10.3402/gha.v8.29034 PMC4576419

[32] Chen J, Zhu Y, Li L, Lv J, Li Z, Chen X, et al. Visual impairment burden in retinopathy of prematurity: trends, inequalities, and improvement gaps. Eur J Pediatr. 2024;183(4):1891–900. 38319404 10.1007/s00431-024-05450-5

[33] Ordunez P, Martinez R, Soliz P, Giraldo G, Mujica OJ, Nordet P. Rheumatic heart disease burden, trends, and inequalities in the Americas, 1990–2017: a population-based study. Lancet Glob Health. 2019;7(10):e1388–97. 31537369 10.1016/S2214-109X(19)30360-2

[34] Cao F, He YS, Wang Y, Zha CK, Lu JM, Tao LM, et al. Global burden and cross-country inequalities in autoimmune diseases from 1990 to 2019. Autoimmun Rev. 2023;22(6):103326. 36958621 10.1016/j.autrev.2023.103326

[35] Ehrlich R, Harris A, Kheradiya NS, Winston DM, Ciulla TA, Wirostko B. Age-related macular degeneration and the aging eye. Clin Interv Aging. 2008;3(3):473–82. 18982917 10.2147/cia.s2777 PMC2682379

[36] Mencucci R, Stefanini S, Favuzza E, Cennamo M, De Vitto C, Mossello E. Beyond vision: cataract and health status in old age, a narrative review. Front Med (Lausanne). 2023;10:1110383. 37007780 10.3389/fmed.2023.1110383 PMC10061098

[37] Zhang Y, Huang S, Xie B, Zhong Y. Aging, cellular senescence, and glaucoma. Aging Dis. 2024;15(2):546–64. 37725658 10.14336/AD.2023.0630-1 PMC10917531

[38] Bloch SB, Larsen M, Munch IC. Incidence of legal blindness from age-related macular degeneration in Denmark: year 2000 to 2010. Am J Ophthalmol. 2012;153(2):209–13.e2. 22264944 10.1016/j.ajo.2011.10.016

[39] Rahman F, Zekite A, Bunce C, Jayaram H, Flanagan D. Recent trends in vision impairment certifications in England and Wales. Eye (Lond). 2020;34(7):1271–8. 32291405 10.1038/s41433-020-0864-6 PMC7314787

[40] Vision Loss Expert Group of the Global Burden of Disease Study, GBD 2019 Blindness and Vision Impairment Collaborators. Global estimates on the number of people blind or visually impaired by age-related macular degeneration: a meta-analysis from 2000 to 2020. Eye (Lond). 2024;38(11):2070–82. 38965321 10.1038/s41433-024-03050-z PMC11269688

[41] McCormick I, Butcher R, Evans JR, Mactaggart IZ, Limburg H, Jolley E, et al. Effective cataract surgical coverage in adults aged 50 years and older: estimates from population-based surveys in 55 countries. Lancet Glob Health. 2022;10(12):e1744–53. 36240806 10.1016/S2214-109X(22)00419-3 PMC7618287

[42] Vision Loss Expert Group of the Global Burden of Disease Study, GBD 2019 Blindness and Vision Impairment Collaborators. Global estimates on the number of people blind or visually impaired by cataract: a meta-analysis from 2000 to 2020. Eye (Lond). 2024;38(11):2156–72. 38461217 10.1038/s41433-024-02961-1 PMC11269584

[43] Bao S, Yang Z, Zhang Z, Qu J, Sun J. AttResAMD: an attention-driven deep learning framework for expert-level automated classification of age-related macular degeneration from fundus photography. Interdiscip Sci. 2025. 10.1007/s12539-025-00763-x. 40885885 10.1007/s12539-025-00763-x

[44] De Fauw J, Ledsam JR, Romera-Paredes B, Nikolov S, Tomasev N, Blackwell S, et al. Clinically applicable deep learning for diagnosis and referral in retinal disease. Nat Med. 2018;24(9):1342–50. 30104768 10.1038/s41591-018-0107-6

[45] Foo VHX, Lim GYS, Liu YC, Ong HS, Wong E, Chan S, et al. Deep learning for detection of Fuchs endothelial dystrophy from widefield specular microscopy imaging: a pilot study. Eye Vis (Lond). 2024;11(1):11. 38494521 10.1186/s40662-024-00378-1 PMC10946096

[46] Hanna A, Martinez DL, Popovic M, Ahmed IIK, Teichman J. Virtual follow-up after cataract surgery: systematic review. J Cataract Refract Surg. 2025;51(2):167–74. 39418044 10.1097/j.jcrs.0000000000001571

[47] Keenan TDL, Chen Q, Agron E, Tham YC, Goh JHL, Lei X, et al. DeepLensNet: deep learning automated diagnosis and quantitative classification of cataract type and severity. Ophthalmology. 2022;129(5):571–84. 34990643 10.1016/j.ophtha.2021.12.017 PMC9038670

[48] Liu H, Li L, Wormstone IM, Qiao C, Zhang C, Liu P, et al. Development and validation of a deep learning system to detect glaucomatous optic neuropathy using fundus photographs. JAMA Ophthalmol. 2019;137(12):1353–60. 31513266 10.1001/jamaophthalmol.2019.3501 PMC6743057

[49] Muhsin ZJ, Qahwaji R, Ghafir I, AlShawabkeh M, Al Bdour M, AlRyalat SA, et al. Highly efficient stacking ensemble learning model for automated keratoconus screening. Eye Vis (Lond). 2025;12(1):25. 40556022 10.1186/s40662-025-00440-6 PMC12186405

[50] Ren Y, Wen H, Bai F, Huang B, Wang Z, Zhang S, et al. Comparison of deep learning-assisted blinking analysis system and Lipiview interferometer in dry eye patients: a cross-sectional study. Eye Vis (Lond). 2024;11(1):7. 38374153 10.1186/s40662-024-00373-6 PMC10875838

[51] Wang S, He X, Jian Z, Li J, Xu C, Chen Y, et al. Advances and prospects of multi-modal ophthalmic artificial intelligence based on deep learning: a review. Eye Vis (Lond). 2024;11(1):38. 39350240 10.1186/s40662-024-00405-1 PMC11443922

[52] Wang Y, Yang Z, Guo X, Jin W, Lin D, Chen A, et al. Automated early detection of acute retinal necrosis from ultra-widefield color fundus photography using deep learning. Eye Vis (Lond). 2024;11(1):27. 39085922 10.1186/s40662-024-00396-z PMC11293155

[53] Yao J, Lim J, Lim GYS, Ong JCL, Ke Y, Tan TF, et al. Novel artificial intelligence algorithms for diabetic retinopathy and diabetic macular edema. Eye Vis (Lond). 2024;11(1):23. 38880890 10.1186/s40662-024-00389-y PMC11181581

[54] Chen SP, Woreta F, Chang DF. Cataracts: a review. JAMA. 2025;333(23):2093-103. 40227658 10.1001/jama.2025.1597

[55] Gupta PC, Ram J. Long-term outcomes of cataract surgery: 15-year results of a prospective study. J Cataract Refract Surg. 2016;42(6):944. 27373403 10.1016/j.jcrs.2016.04.013

[56] Jayaram H, Kolko M, Friedman DS, Gazzard G. Glaucoma: now and beyond. Lancet. 2023;402(10414):1788–801. 37742700 10.1016/S0140-6736(23)01289-8

[57] Schultz NM, Bhardwaj S, Barclay C, Gaspar L, Schwartz J. Global burden of dry age-related macular degeneration: a targeted literature review. Clin Ther. 2021;43(10):1792–818. 34548176 10.1016/j.clinthera.2021.08.011

[58] Jiménez-Gómez Y, Alba-Molina D, Blanco-Blanco M, Pérez-Fajardo L, Reyes-Ortega F, Ortega-Llamas L, et al. Novel treatments for age-related macular degeneration: a review of clinical advances in sustained drug delivery systems. Pharmaceutics. 2022;14(7):1473. 35890368 10.3390/pharmaceutics14071473 PMC9319243

[59] Gayraud L, Mortamais M, Schweitzer C, de Hoogh K, Cougnard-Grégoire A, Korobelnik JF, et al. Ambient air pollution exposure and incidence of cataract surgery: the prospective 3City-Alienor study. Acta Ophthalmol. 2025;103(3):e192–9. 39528362 10.1111/aos.16790 PMC11986394

[60] Ravilla TD, Gupta S, Ravindran RD, Vashist P, Krishnan T, Maraini G, et al. Use of cooking fuels and cataract in a population-based study: the India Eye Disease Study. Environ Health Perspect. 2016;124(12):1857–62. 27227523 10.1289/EHP193 PMC5132636

[61] Nuzzi R, Caselgrandi P. Sex hormones and their effects on ocular disorders and pathophysiology: current aspects and our experience. Int J Mol Sci. 2022;23(6):3269. 35328690 10.3390/ijms23063269 PMC8949880

[62] Wilkinson J, Mailu EW, Virendrakumar B, Bechange S, Jolley E, Schmidt E. Factors associated with the uptake of cataract surgery and interventions to improve uptake in low- and middle-income countries: a systematic review. PLoS One. 2020;15(7):e0235699. 32645065 10.1371/journal.pone.0235699 PMC7347115

[63] Bechange S, Jolley E, Virendrakumar B, Pente V, Milgate J, Schmidt E. Strengths and weaknesses of eye care services in sub-Saharan Africa: a meta-synthesis of eye health system assessments. BMC Health Serv Res. 2020;20(1):381. 32375761 10.1186/s12913-020-05279-2 PMC7203845

[64] Foreman J, Keel S, van Wijngaarden P, Bourne RA, Wormald R, Crowston J, et al. Prevalence and causes of visual loss among the indigenous peoples of the world: a systematic review. JAMA Ophthalmol. 2018;136(5):567–80. 29596691 10.1001/jamaophthalmol.2018.0597

[65] Davis A, Lembo T, Laurie E, Mutua E, Loosli K, Nthambi M, et al. How public health crises expose systemic, day-to-day health inequalities in low-and-middle income countries: an example from East Africa. Antimicrob Resist Infect Control. 2022;11(1):34. 35164886 10.1186/s13756-022-01071-5 PMC8842514

[66] Jayatilleke K. Challenges in implementing surveillance tools of high-income countries (HICs) in low middle income countries (LMICs). Curr Treat Options Infect Dis. 2020;12(3):191–201. 32874140 10.1007/s40506-020-00229-2 PMC7453076

