# Supplementary material for: Global burden and cross-country inequalities of age-related eye diseases from 1990 to 2021: a comprehensive analysis of temporal trends and socioeconomic disparities
Source: Eye Vis (Lond). 2026 Feb 1;13:4. doi: 10.1186/s40662-026-00473-5 (PMC12861071; doi:10.1186/s40662-026-00473-5)
Supplement: Supplementary file 1 — Supplementary material 1. [file 40662_2026_473_MOESM1_ESM.docx]

**Supplementary Materials**

**Supplementary Figure S1. Temporal trends in absolute inequalities (slope index of inequality [SII]) for age-related eye diseases across Global Burden of Disease regions.**

**
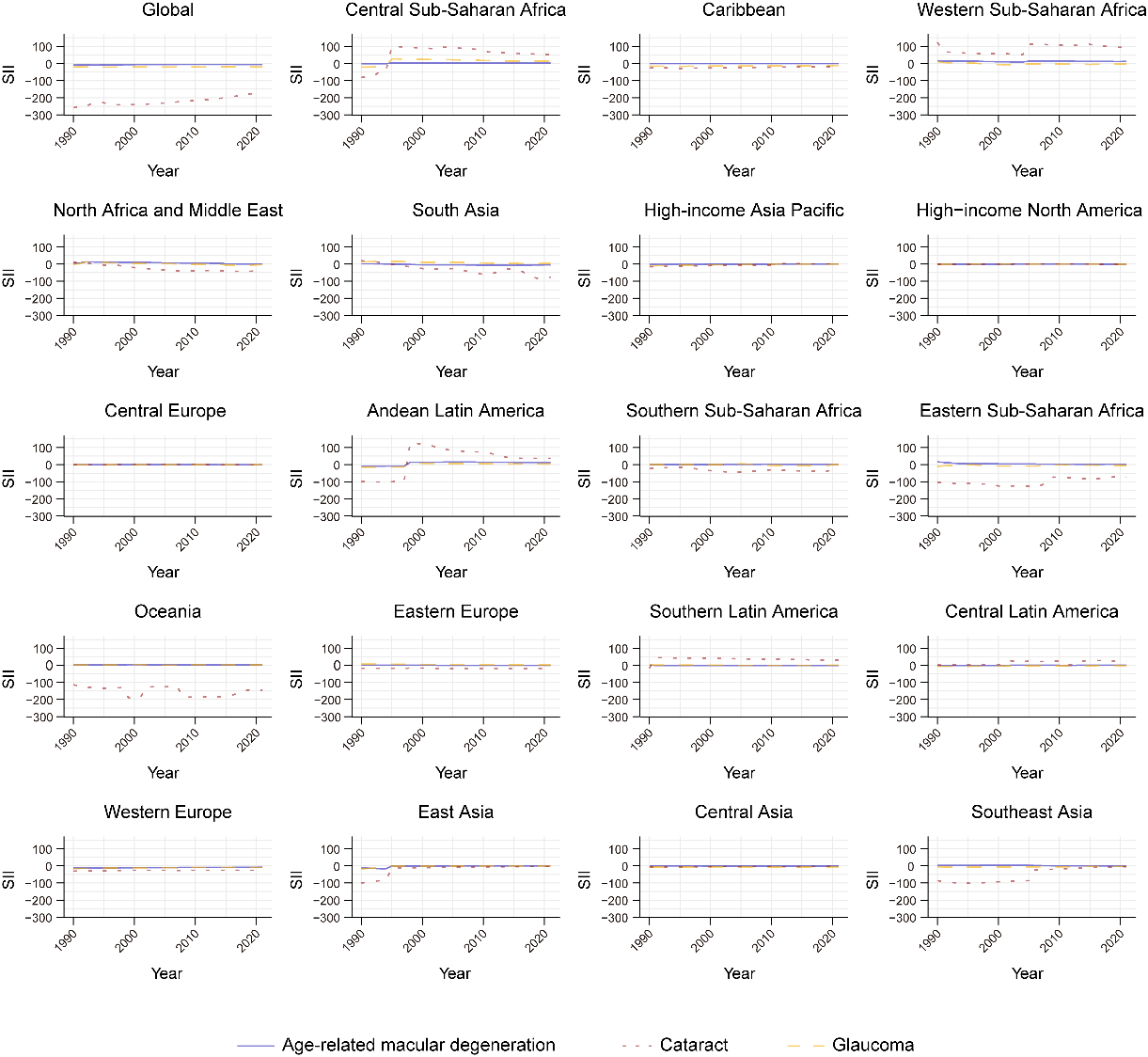
**

**Supplementary Figure S2. Temporal trends in relative inequalities (concentration index [CI]) for age-related eye diseases across Global Burden of Disease regions.**


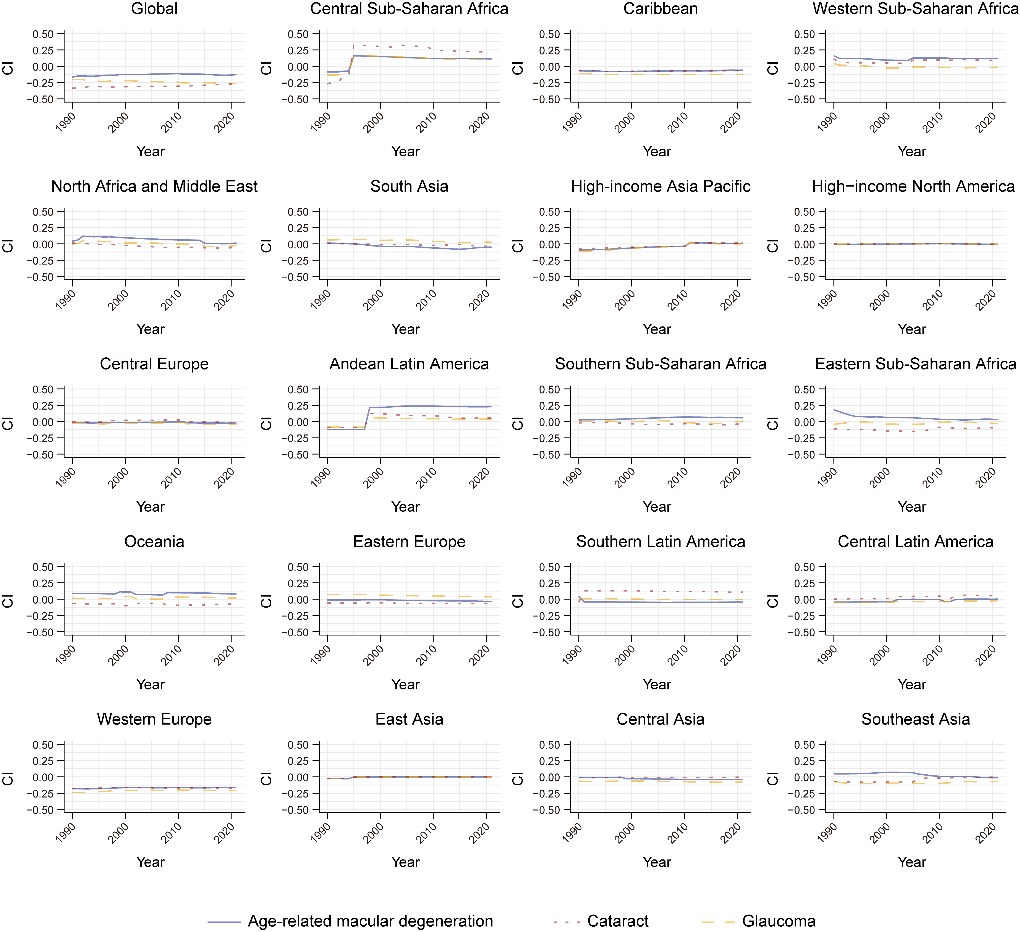


**Supplementary Table S1. Global trends in YLD rates of age-related eye diseases from 1990 to 2021.**

| Disease | Time period information | | APC (95% CI) | *P* value |
| --- | --- | --- | --- | --- |
| Age-related macular degeneration | Period 1 | 1990–1993 | 2.441 (2.141 to 2.742) | < 0.001 |
|  | Period 2 | 1993–1996 | 0.648 (0.063 to 1.236) | 0.033 |
|  | Period 3 | 1996–1999 | −1.474 (−2.040 to −0.905) | < 0.001 |
|  | Period 4 | 1999–2006 | 0.741 (0.644 to 0.838) | < 0.001 |
|  | Period 5 | 2006–2009 | −0.032 (−0.593 to 0.532) | 0.904 |
|  | Period 6 | 2009–2018 | 0.935 (0.873 to 0.997) | < 0.001 |
|  | Period 7 | 2018–2021 | 2.426 (2.138 to 2.716) | < 0.001 |
| Cataract | Period 1 | 1990–1995 | 0.289 (0.021 to 0.558) | 0.036 |
|  | Period 2 | 1995–2001 | 1.943 (1.673 to 2.214) | < 0.001 |
|  | Period 3 | 2001–2010 | 0.514 (0.384 to 0.645) | < 0.001 |
|  | Period 4 | 2010–2015 | 1.237 (0.851 to 1.624) | < 0.001 |
|  | Period 5 | 2015–2018 | −0.108 (−1.319 to 1.118) | 0.853 |
|  | Period 6 | 2018–2021 | 1.175 (0.557 to 1.797) | 0.001 |
| Glaucoma | Period 1 | 1990–1994 | −0.747 (−0.856 to −0.638) | < 0.001 |
|  | Period 2 | 1994–2001 | 0.562 (0.504 to 0.621) | < 0.001 |
|  | Period 3 | 2001–2010 | −0.046 (−0.083 to −0.008) | 0.021 |
|  | Period 4 | 2010–2016 | 0.844 (0.767 to 0.922) | < 0.001 |
|  | Period 5 | 2016–2019 | 1.223 (0.877 to 1.571) | < 0.001 |
|  | Period 6 | 2019–2021 | 0.282 (−0.060 to 0.626) | 0.099 |

YLDs = years lived with disability; APC = annual percent change; CI = confidence interval

**Supplementary Table S2. Global trends in ASYR of age-related eye diseases from 1990 to 2021.**

| Disease | Time period information | | APC (95% CI) | *P* value |
| --- | --- | --- | --- | --- |
| Age-related macular degeneration | Period 1 | 1990–1993 | 1.306 (1.041 to 1.572) | < 0.001 |
|  | Period 2 | 1993–1996 | −0.396 (−0.917 to 0.128) | 0.125 |
|  | Period 3 | 1996–1999 | −2.487 (−2.994 to −1.978) | < 0.001 |
|  | Period 4 | 1999–2006 | −0.605 (−0.692 to −0.519) | < 0.001 |
|  | Period 5 | 2006–2009 | −1.627 (−2.129 to −1.121) | < 0.001 |
|  | Period 6 | 2009–2018 | −0.949 (−1.004 to −0.894) | < 0.001 |
|  | Period 7 | 2018–2021 | 0.410 (0.154 to 0.666) | 0.004 |
| Cataract | Period 1 | 1990–1995 | −0.688 (−0.961 to −0.414) | < 0.001 |
|  | Period 2 | 1995–2001 | 0.807 (0.529 to 1.086) | < 0.001 |
|  | Period 3 | 2001–2011 | −0.862 (−0.975 to −0.749) | < 0.001 |
|  | Period 4 | 2011–2015 | −0.345 (−0.975 to 0.289) | 0.264 |
|  | Period 5 | 2015–2018 | −1.893 (−3.140 to −0.629) | 0.006 |
|  | Period 6 | 2018–2021 | −0.516 (−1.154 to 0.127) | 0.107 |
| Glaucoma | Period 1 | 1990–1994 | −1.768 (−1.831 to −1.705) | < 0.001 |
|  | Period 2 | 1994–2001 | −0.689 (−0.723 to −0.654) | < 0.001 |
|  | Period 3 | 2001–2006 | −1.489 (−1.552 to −1.425) | < 0.001 |
|  | Period 4 | 2006–2010 | −1.776 (−1.877 to −1.676) | < 0.001 |
|  | Period 5 | 2010–2019 | −1.005 (−1.027 to −0.983) | < 0.001 |
|  | Period 6 | 2019–2021 | −1.411 (−1.612 to −1.210) | < 0.001 |

ASYR = age-standardized of years lived with disability rate; APC = annual percent change; CI = confidence interval

**Supplementary Table S3. National/regional burden of age-related macular degeneration in 1990 and 2021 with AAPC.**

| Country/Region | ASYR (95% UI) | | AAPC  (95% CI) | *P* value |
| --- | --- | --- | --- | --- |
|  | 1990 | 2021 |  |  |
| Afghanistan | 20.549  (13.787 to 29.329) | 19.871  (13.360 to 28.383) | −0.103  (−0.142 to −0.064) | < 0.001 |
| Albania | 5.359  (3.504 to 7.584) | 4.421  (2.966 to 6.070) | −0.646  (−0.725 to −0.566) | < 0.001 |
| Algeria | 17.070  (11.362 to 24.278) | 13.228  (9.030 to 18.769) | −0.813  (−0.852 to −0.774) | < 0.001 |
| American Samoa | 3.940  (2.533 to 5.898) | 2.751  (1.764 to 4.057) | −1.158  (−1.235 to −1.081) | < 0.001 |
| Andorra | 8.349  (5.483 to 11.462) | 6.698  (4.498 to 9.175) | −0.705  (−0.759 to −0.652) | < 0.001 |
| Angola | 3.430  (2.241 to 4.949) | 2.880  (1.912 to 4.045) | −0.570  (−0.603 to −0.537) | < 0.001 |
| Antigua and Barbuda | 1.773  (1.172 to 2.474) | 1.407  (0.914 to 2.021) | −0.730  (−0.766 to −0.694) | < 0.001 |
| Argentina | 3.960  (2.499 to 5.664) | 2.973  (1.928 to 4.106) | −0.920  (−0.974 to −0.866) | < 0.001 |
| Armenia | 4.842  (3.247 to 6.762) | 4.399  (2.930 to 6.128) | −0.301  (−0.345 to −0.257) | < 0.001 |
| Australia | 4.683  (3.068 to 6.655) | 3.645  (2.358 to 5.154) | −0.805  (−0.862 to −0.748) | < 0.001 |
| Austria | 9.441  (6.203 to 12.982) | 6.961  (4.657 to 9.434) | −0.983  (−1.028 to −0.937) | < 0.001 |
| Azerbaijan | 5.172  (3.431 to 7.371) | 4.643  (3.130 to 6.465) | −0.330  (−0.431 to −0.229) | < 0.001 |
| Bahamas | 1.809  (1.151 to 2.550) | 1.468  (0.967 to 2.090) | −0.674  (−0.750 to −0.599) | < 0.001 |
| Bahrain | 16.419  (10.951 to 23.123) | 11.459  (7.928 to 16.244) | −1.150  (−1.186 to −1.113) | < 0.001 |
| Bangladesh | 9.185  (6.283 to 13.032) | 6.493  (4.516 to 9.052) | −1.028  (−1.156 to −0.899) | < 0.001 |
| Barbados | 0.596  (0.396 to 0.871) | 0.531  (0.347 to 0.765) | −0.371  (−0.446 to −0.295) | < 0.001 |
| Belarus | 2.246  (1.434 to 3.227) | 1.847  (1.169 to 2.607) | −0.601  (−0.668 to −0.535) | < 0.001 |
| Belgium | 9.472  (6.275 to 12.734) | 7.146  (4.713 to 9.729) | −0.911  (−0.942 to −0.880) | < 0.001 |
| Belize | 1.953  (1.265 to 2.757) | 1.576  (1.034 to 2.252) | −0.682  (−0.774 to −0.590) | < 0.001 |
| Benin | 8.425  (5.883 to 11.966) | 13.470  (9.385 to 18.798) | 1.526  (1.455 to 1.596) | < 0.001 |
| Bermuda | 1.667  (1.087 to 2.356) | 1.271  (0.845 to 1.814) | −0.863  (−0.914 to −0.812) | < 0.001 |
| Bhutan | 5.139  (3.480 to 7.285) | 3.601  (2.406 to 5.044) | −1.151  (−1.257 to −1.045) | < 0.001 |
| Bolivia | 6.761  (4.526 to 9.523) | 5.480  (3.728 to 7.726) | −0.658  (−0.676 to −0.641) | < 0.001 |
| Bosnia and Herzegovina | 5.078  (3.344 to 7.188) | 4.022  (2.705 to 5.640) | −0.764  (−0.954 to −0.574) | < 0.001 |
| Botswana | 2.924  (1.802 to 4.267) | 2.195  (1.344 to 3.285) | −0.934  (−1.011 to −0.857) | < 0.001 |
| Brazil | 4.884  (3.440 to 6.685) | 4.480  (3.157 to 6.148) | −0.275  (−0.365 to −0.185) | < 0.001 |
| Brunei Darussalam | 2.874  (1.843 to 4.055) | 2.239  (1.468 to 3.144) | −0.795  (−0.857 to −0.734) | < 0.001 |
| Bulgaria | 4.061  (2.868 to 5.428) | 3.374  (2.248 to 4.805) | −0.598  (−0.680 to −0.516) | < 0.001 |
| Burkina Faso | 4.916  (3.257 to 7.036) | 7.757  (5.213 to 11.226) | 1.543  (1.322 to 1.765) | < 0.001 |
| Burundi | 6.586  (4.192 to 9.429) | 5.379  (3.530 to 7.754) | −0.657  (−0.723 to −0.591) | < 0.001 |
| Cabo Verde | 12.548  (8.997 to 17.277) | 10.185  (6.985 to 14.631) | −0.675  (−0.738 to −0.612) | < 0.001 |
| Cambodia | 7.682  (4.827 to 11.244) | 5.441  (3.613 to 7.880) | −1.099  (−1.188 to −1.010) | < 0.001 |
| Cameroon | 10.136  (6.989 to 14.239) | 9.949  (6.933 to 13.952) | −0.062  (−0.115 to −0.009) | 0.021 |
| Canada | 2.708  (1.789 to 3.863) | 2.435  (1.598 to 3.351) | −0.337  (−0.393 to −0.282) | < 0.001 |
| Central African Republic | 1.592  (1.053 to 2.244) | 1.756  (1.103 to 2.535) | 0.309  (0.253 to 0.365) | < 0.001 |
| Chad | 8.348  (5.604 to 11.709) | 10.633  (7.467 to 14.997) | 0.776  (0.715 to 0.837) | < 0.001 |
| Chile | 3.047  (1.970 to 4.264) | 2.343  (1.514 to 3.319) | −0.832  (−0.887 to −0.777) | < 0.001 |
| China | 7.451  (5.253 to 10.324) | 7.247  (5.039 to 9.97) | −0.082  (−0.204 to 0.040) | 0.186 |
| Colombia | 4.684  (3.191 to 6.637) | 3.585  (2.392 to 5.102) | −0.861  (−0.893 to −0.828) | < 0.001 |
| Comoros | 13.450  (8.774 to 19.442) | 10.127  (6.774 to 14.654) | −0.907  (−0.933 to −0.880) | < 0.001 |
| Congo | 3.117  (2.093 to 4.338) | 2.659  (1.758 to 3.731) | −0.519  (−0.608 to −0.429) | < 0.001 |
| Cook Islands | 6.683  (4.176 to 9.867) | 4.178  (2.592 to 6.027) | −1.505  (−1.627 to −1.383) | < 0.001 |
| Costa Rica | 3.074  (1.970 to 4.441) | 2.508  (1.636 to 3.514) | −0.644  (−0.694 to −0.594) | < 0.001 |
| Croatia | 4.376  (2.862 to 6.162) | 3.779  (2.537 to 5.275) | −0.502  (−0.652 to −0.352) | < 0.001 |
| Cuba | 2.289  (1.502 to 3.267) | 1.852  (1.227 to 2.660) | −0.678  (−0.809 to −0.547) | < 0.001 |
| Cyprus | 9.873  (6.521 to 13.646) | 6.824  (4.584 to 9.306) | −1.184  (−1.239 to −1.130) | < 0.001 |
| Czechia | 4.412  (2.989 to 6.169) | 3.553  (2.396 to 4.981) | −0.692  (−0.791 to −0.592) | < 0.001 |
| Democratic People's Republic of Korea | 2.470  (1.659 to 3.574) | 1.972  (1.300 to 2.978) | −0.720  (−0.795 to −0.646) | < 0.001 |
| Democratic Republic of the Congo | 1.520  (1.006 to 2.208) | 1.699  (1.153 to 2.469) | 0.376  (0.315 to 0.438) | < 0.001 |
| Denmark | 6.196  (4.159 to 8.620) | 4.974  (3.308 to 6.949) | −0.709  (−0.757 to −0.660) | < 0.001 |
| Djibouti | 13.272  (8.610 to 19.349) | 9.893  (6.622 to 14.180) | −0.943  (−0.981 to −0.906) | < 0.001 |
| Dominica | 1.904  (1.211 to 2.747) | 1.507  (0.981 to 2.171) | −0.724  (−0.758 to −0.690) | < 0.001 |
| Dominican Republic | 2.960  (1.792 to 4.382) | 2.191  (1.388 to 3.254) | −0.911  (−1.061 to −0.761) | < 0.001 |
| Ecuador | 2.727  (1.832 to 3.856) | 2.270  (1.495 to 3.237) | −0.573  (−0.615 to −0.532) | < 0.001 |
| Egypt | 13.939  (9.273 to 20.113) | 13.922  (9.611 to 19.671) | −0.017  (−0.110 to 0.077) | 0.725 |
| El Salvador | 7.211  (4.656 to 10.343) | 5.006  (3.339 to 7.024) | −1.166  (−1.242 to −1.089) | < 0.001 |
| Equatorial Guinea | 3.447  (2.209 to 5.122) | 2.666  (1.727 to 3.833) | −0.825  (−0.899 to −0.751) | < 0.001 |
| Eritrea | 13.286  (8.640 to 18.948) | 10.304  (6.833 to 14.411) | −0.790  (−0.875 to −0.705) | < 0.001 |
| Estonia | 1.514  (1.015 to 2.159) | 1.214  (0.802 to 1.765) | −0.716  (−0.772 to −0.660) | < 0.001 |
| Eswatini | 3.172  (2.070 to 4.582) | 2.775  (1.797 to 3.931) | −0.437  (−0.458 to −0.415) | < 0.001 |
| Ethiopia | 10.873  (7.463 to 15.684) | 12.833  (8.644 to 18.058) | 0.526  (0.454 to 0.598) | < 0.001 |
| Fiji | 5.684  (3.651 to 8.489) | 4.336  (2.766 to 6.411) | −0.908  (−1.100 to −0.716) | < 0.001 |
| Finland | 10.688  (7.095 to 14.695) | 7.065  (4.694 to 9.574) | −1.333  (−1.378 to −1.288) | < 0.001 |
| France | 8.568  (5.710 to 11.587) | 6.309  (4.250 to 8.487) | −0.979  (−1.019 to −0.940) | < 0.001 |
| Gabon | 3.091  (1.993 to 4.381) | 2.526  (1.627 to 3.551) | −0.655  (−0.717 to −0.592) | < 0.001 |
| Gambia | 7.968  (5.562 to 11.126) | 9.424  (6.594 to 13.162) | 0.529  (0.409 to 0.650) | < 0.001 |
| Georgia | 4.755  (3.230 to 6.646) | 4.583  (3.059 to 6.365) | −0.102  (−0.177 to −0.027) | 0.008 |
| Germany | 9.089  (6.068 to 12.526) | 6.408  (4.232 to 8.793) | −1.124  (−1.158 to −1.091) | < 0.001 |
| Ghana | 8.515  (5.903 to 12.051) | 11.162  (7.732 to 15.658) | 0.858  (0.700 to 1.016) | < 0.001 |
| Greece | 8.888  (5.831 to 12.204) | 6.923  (4.566 to 9.753) | −0.806  (−0.854 to −0.758) | < 0.001 |
| Greenland | 3.738  (2.441 to 5.393) | 3.180  (2.128 to 4.435) | −0.528  (−0.606 to −0.450) | < 0.001 |
| Grenada | 1.959  (1.269 to 2.759) | 1.496  (0.976 to 2.109) | −0.841  (−0.898 to −0.783) | < 0.001 |
| Guam | 3.747  (2.386 to 5.601) | 3.082  (2.002 to 4.578) | −0.631  (−0.694 to −0.568) | < 0.001 |
| Guatemala | 6.01  (3.877 to 8.614) | 4.355  (2.822 to 6.142) | −1.018  (−1.079 to −0.957) | < 0.001 |
| Guinea | 9.758  (6.678 to 13.542) | 11.969  (8.261 to 16.579) | 0.649  (0.610 to 0.687) | < 0.001 |
| Guinea−Bissau | 11.420  (7.937 to 15.552) | 14.232  (9.891 to 20.150) | 0.779  (0.627 to 0.931) | < 0.001 |
| Guyana | 2.064  (1.354 to 2.943) | 1.591  (1.038 to 2.260) | −0.805  (−0.856 to −0.754) | < 0.001 |
| Haiti | 2.406  (1.541 to 3.407) | 1.912  (1.262 to 2.704) | −0.709  (−0.751 to −0.668) | < 0.001 |
| Honduras | 5.451  (3.567 to 7.779) | 4.217  (2.821 to 5.972) | −0.811  (−0.921 to −0.701) | < 0.001 |
| Hungary | 6.032  (3.989 to 8.486) | 4.969  (3.341 to 6.823) | −0.641  (−0.895 to −0.386) | < 0.001 |
| Iceland | 19.865  (13.558 to 27.015) | 11.812  (7.836 to 16.214) | −1.689  (−1.751 to −1.627) | < 0.001 |
| India | 13.232  (8.782 to 18.977) | 7.878  (5.374 to 11.135) | −1.665  (−1.757 to −1.574) | < 0.001 |
| Indonesia | 7.473  (4.422 to 11.713) | 5.308  (3.242 to 7.978) | −1.093  (−1.115 to −1.070) | < 0.001 |
| Iran (Islamic Republic of) | 30.065  (20.519 to 41.873) | 25.021  (17.157 to 34.884) | −0.567  (−0.638 to −0.497) | < 0.001 |
| Iraq | 17.332  (11.711 to 24.346) | 13.178  (9.118 to 18.376) | −0.890  (−0.932 to −0.848) | < 0.001 |
| Ireland | 9.606  (6.445 to 13.074) | 6.869  (4.612 to 9.385) | −1.093  (−1.140 to −1.046) | < 0.001 |
| Israel | 9.663  (6.456 to 13.239) | 7.391  (4.969 to 10.004) | −0.867  (−0.906 to −0.829) | < 0.001 |
| Italy | 20.854  (13.586 to 28.739) | 13.092  (8.689 to 18.109) | −1.488  (−1.574 to −1.401) | < 0.001 |
| Ivory Coast | 4.687  (3.286 to 6.581) | 11.272  (7.848 to 15.676) | 2.852  (2.647 to 3.058) | < 0.001 |
| Jamaica | 1.810  (1.177 to 2.619) | 1.468  (0.929 to 2.071) | −0.659  (−0.700 to −0.617) | < 0.001 |
| Japan | 2.342  (1.512 to 3.278) | 1.938  (1.274 to 2.657) | −0.603  (−0.647 to −0.559) | < 0.001 |
| Jordan | 20.188  (13.224 to 29.431) | 12.620  (8.369 to 18.277) | −1.489  (−1.56 to −1.419) | < 0.001 |
| Kazakhstan | 5.018  (3.443 to 6.892) | 4.579  (3.074 to 6.416) | −0.282  (−0.351 to −0.213) | < 0.001 |
| Kenya | 34.319  (22.138 to 49.319) | 15.377  (9.889 to 22.138) | −2.529  (−2.661 to −2.397) | < 0.001 |
| Kiribati | 4.735  (2.960 to 7.064) | 3.913  (2.524 to 5.737) | −0.607  (−0.697 to −0.517) | < 0.001 |
| Kuwait | 14.021  (9.503 to 19.499) | 10.823  (7.323 to 14.858) | −0.826  (−0.879 to −0.773) | < 0.001 |
| Kyrgyzstan | 7.921  (5.399 to 11.217) | 7.736  (5.256 to 10.985) | −0.051  (−0.102 to 0.000) | 0.051 |
| Lao People's Democratic Republic | 5.797  (3.910 to 8.257) | 4.653  (3.114 to 6.620) | −0.699  (−0.763 to −0.636) | < 0.001 |
| Latvia | 2.195  (1.399 to 3.132) | 1.843  (1.199 to 2.597) | −0.560  (−0.659 to −0.461) | < 0.001 |
| Lebanon | 15.897  (11.173 to 21.824) | 12.948  (8.620 to 18.223) | −0.660  (−0.733 to −0.586) | < 0.001 |
| Lesotho | 3.300  (2.111 to 4.778) | 3.002  (1.941 to 4.416) | −0.306  (−0.393 to −0.218) | < 0.001 |
| Liberia | 10.993  (7.722 to 15.399) | 9.857  (6.875 to 13.843) | −0.370  (−0.413 to −0.326) | < 0.001 |
| Libya | 12.712  (8.581 to 18.280) | 10.085  (6.834 to 14.224) | −0.855  (−1.144 to −0.565) | < 0.001 |
| Lithuania | 2.184  (1.405 to 3.149) | 1.83  (1.137 to 2.627) | −0.557  (−0.666 to −0.449) | < 0.001 |
| Luxembourg | 9.567  (6.284 to 12.972) | 6.622  (4.407 to 9.162) | −1.188  (−1.211 to −1.166) | < 0.001 |
| Madagascar | 8.637  (5.675 to 12.316) | 6.588  (4.355 to 9.381) | −0.863  (−0.898 to −0.828) | < 0.001 |
| Malawi | 14.554  (9.778 to 20.859) | 11.816  (7.710 to 16.940) | −0.671  (−0.730 to −0.613) | < 0.001 |
| Malaysia | 21.405  (14.210 to 30.337) | 10.763  (7.416 to 15.625) | −2.194  (−2.255 to −2.133) | < 0.001 |
| Maldives | 10.646  (7.092 to 14.996) | 6.462  (4.352 to 9.275) | −1.603  (−1.669 to −1.536) | < 0.001 |
| Mali | 9.314  (6.491 to 12.442) | 8.236  (5.540 to 11.865) | −0.419  (−0.484 to −0.354) | < 0.001 |
| Malta | 9.701  (6.291 to 13.274) | 6.996  (4.622 to 9.505) | −1.053  (−1.087 to −1.019) | < 0.001 |
| Marshall Islands | 4.690  (2.971 to 6.917) | 3.188  (2.088 to 4.655) | −1.243  (−1.341 to −1.144) | < 0.001 |
| Mauritania | 11.780  (8.232 to 16.393) | 10.858  (7.608 to 15.606) | −0.271  (−0.282 to −0.260) | < 0.001 |
| Mauritius | 8.243  (5.604 to 11.727) | 5.855  (3.984 to 8.457) | −1.091  (−1.126 to −1.055) | < 0.001 |
| Mexico | 5.218  (3.583 to 7.184) | 4.056  (2.792 to 5.562) | −0.816  (−0.855 to −0.778) | < 0.001 |
| Micronesia (Federated States of) | 4.888  (3.073 to 7.033) | 3.612  (2.281 to 5.412) | −0.982  (−1.058 to −0.906) | < 0.001 |
| Monaco | 8.565  (5.710 to 11.681) | 6.930  (4.631 to 9.441) | −0.69  (−0.735 to −0.644) | < 0.001 |
| Mongolia | 4.418  (2.943 to 6.181) | 3.993  (2.558 to 5.687) | −0.302  (−0.388 to −0.215) | < 0.001 |
| Montenegro | 4.261  (2.869 to 6.034) | 3.874  (2.628 to 5.413) | −0.301  (−0.508 to −0.094) | 0.004 |
| Morocco | 9.742  (7.089 to 13.130) | 8.578  (5.674 to 12.130) | −0.417  (−0.447 to −0.387) | < 0.001 |
| Mozambique | 16.957  (10.938 to 24.686) | 13.492  (8.946 to 19.147) | −0.733  (−0.848 to −0.619) | < 0.001 |
| Myanmar | 5.966  (3.868 to 8.933) | 4.772  (3.214 to 6.907) | −0.715  (−0.803 to −0.627) | < 0.001 |
| Namibia | 3.138  (2.056 to 4.516) | 2.637  (1.709 to 3.842) | −0.576  (−0.601 to −0.551) | < 0.001 |
| Nauru | 4.355  (2.728 to 6.622) | 3.607  (2.264 to 5.216) | −0.601  (−0.656 to −0.546) | < 0.001 |
| Nepal | 17.441  (12.442 to 24.903) | 20.274  (13.834 to 29.600) | 0.446  (0.295 to 0.597) | < 0.001 |
| Netherlands | 7.985  (5.283 to 11.050) | 5.844  (3.916 to 8.018) | −1.004  (−1.034 to −0.975) | < 0.001 |
| New Zealand | 5.532  (3.694 to 7.791) | 4.176  (2.827 to 5.847) | −0.912  (−0.958 to −0.866) | < 0.001 |
| Nicaragua | 5.000  (3.217 to 7.153) | 3.842  (2.598 to 5.344) | −0.851  (−0.881 to −0.821) | < 0.001 |
| Niger | 8.480  (5.721 to 12.129) | 12.267  (8.460 to 17.298) | 1.197  (1.084 to 1.309) | < 0.001 |
| Nigeria | 22.185  (15.846 to 30.519) | 19.647  (13.930 to 27.588) | −0.416  (−0.538 to −0.294) | < 0.001 |
| Niue | 4.308  (2.713 to 6.416) | 2.958  (1.926 to 4.367) | −1.208  (−1.295 to −1.122) | < 0.001 |
| North Macedonia | 4.930  (3.272 to 7.009) | 4.038  (2.664 to 5.611) | −0.679  (−0.871 to −0.487) | < 0.001 |
| Northern Mariana Islands | 3.696  (2.305 to 5.353) | 2.950  (1.919 to 4.360) | −0.722  (−0.858 to −0.587) | < 0.001 |
| Norway | 7.316  (4.985 to 9.828) | 6.256  (4.306 to 8.454) | −0.505  (−0.533 to −0.477) | < 0.001 |
| Oman | 18.067  (11.911 to 26.555) | 17.854  (12.007 to 25.379) | −0.030  (−0.119 to 0.058) | 0.502 |
| Pakistan | 14.910  (10.451 to 21.357) | 11.687  (8.237 to 16.343) | −0.790  (−0.872 to −0.709) | < 0.001 |
| Palau | 4.155  (2.574 to 6.110) | 2.979  (1.938 to 4.323) | −1.079  (−1.136 to −1.022) | < 0.001 |
| Palestine | 12.795  (8.733 to 18.186) | 9.353  (6.470 to 13.077) | −1.034  (−1.097 to −0.971) | < 0.001 |
| Panama | 4.695  (3.027 to 7.086) | 3.695  (2.359 to 5.271) | −0.775  (−0.865 to −0.684) | < 0.001 |
| Papua New Guinea | 2.974  (1.926 to 4.363) | 2.460  (1.623 to 3.579) | −0.610  (−0.758 to −0.462) | < 0.001 |
| Paraguay | 3.405  (2.185 to 4.816) | 2.868  (1.864 to 4.100) | −0.559  (−0.616 to −0.502) | < 0.001 |
| Peru | 12.831  (8.785 to 18.088) | 11.351  (7.766 to 15.929) | −0.395  (−0.430 to −0.361) | < 0.001 |
| Philippines | 7.655  (5.272 to 10.724) | 7.129  (4.892 to 10.014) | −0.193  (−0.243 to −0.144) | < 0.001 |
| Poland | 4.328  (3.009 to 6.016) | 3.963  (2.721 to 5.475) | −0.300  (−0.404 to −0.195) | < 0.001 |
| Portugal | 10.805  (7.178 to 14.793) | 7.233  (4.762 to 9.970) | −1.291  (−1.328 to −1.255) | < 0.001 |
| Puerto Rico | 1.635  (1.042 to 2.333) | 1.250  (0.821 to 1.770) | −0.837  (−0.872 to −0.802) | < 0.001 |
| Qatar | 13.699  (9.421 to 19.040) | 9.712  (6.636 to 13.699) | −1.048  (−1.472 to −0.622) | < 0.001 |
| Republic of Korea | 3.703  (2.264 to 5.208) | 2.157  (1.405 to 3.086) | −1.754  (−1.825 to −1.683) | < 0.001 |
| Republic of Moldova | 3.560  (2.281 to 5.129) | 3.128  (2.158 to 4.312) | −0.412  (−0.484 to −0.339) | < 0.001 |
| Romania | 5.114  (3.413 to 7.222) | 4.289  (2.897 to 5.99) | −0.588  (−0.759 to −0.417) | < 0.001 |
| Russian Federation | 2.341  (1.542 to 3.268) | 1.861  (1.234 to 2.585) | −0.733  (−0.785 to −0.680) | < 0.001 |
| Rwanda | 11.214  (7.207 to 16.339) | 7.326  (4.904 to 10.380) | −1.363  (−1.439 to −1.287) | < 0.001 |
| Saint Kitts and Nevis | 1.984  (1.270 to 2.823) | 1.474  (0.974 to 2.107) | −0.943  (−1.000 to −0.887) | < 0.001 |
| Saint Lucia | 1.861  (1.224 to 2.658) | 1.418  (0.955 to 2.024) | −0.859  (−0.902 to −0.816) | < 0.001 |
| Saint Vincent and the Grenadines | 1.918  (1.268 to 2.730) | 1.512  (0.954 to 2.146) | −0.739  (−0.825 to −0.654) | < 0.001 |
| Samoa | 4.446  (2.861 to 6.522) | 3.304  (2.077 to 4.869) | −0.958  (−1.054 to −0.861) | < 0.001 |
| San Marino | 8.326  (5.473 to 11.324) | 6.805  (4.522 to 9.358) | −0.666  (−0.700 to −0.631) | < 0.001 |
| Sao Tome and Principe | 12.497  (8.736 to 17.251) | 11.031  (7.775 to 15.446) | −0.405  (−0.438 to −0.372) | < 0.001 |
| Saudi Arabia | 28.995  (19.021 to 41.152) | 19.224  (13.089 to 26.789) | −1.312  (−1.357 to −1.267) | < 0.001 |
| Senegal | 10.121  (6.707 to 14.230) | 10.684  (7.299 to 15.137) | 0.177  (0.113 to 0.240) | < 0.001 |
| Serbia | 4.837  (3.210 to 6.879) | 3.990  (2.647 to 5.563) | −0.655  (−0.821 to −0.488) | < 0.001 |
| Seychelles | 8.686  (5.794 to 12.427) | 6.174  (4.223 to 8.670) | −1.093  (−1.152 to −1.034) | < 0.001 |
| Sierra Leone | 7.776  (5.319 to 11.018) | 7.305  (5.025 to 10.297) | −0.209  (−0.298 to −0.119) | < 0.001 |
| Singapore | 3.084  (1.954 to 4.403) | 2.560  (1.650 to 3.659) | −0.583  (−0.645 to −0.521) | < 0.001 |
| Slovakia | 4.639  (3.135 to 6.420) | 4.066  (2.767 to 5.800) | −0.421  (−0.567 to −0.275) | < 0.001 |
| Slovenia | 4.229  (2.876 to 5.986) | 3.640  (2.452 to 5.094) | −0.480  (−0.674 to −0.287) | < 0.001 |
| Solomon Islands | 4.566  (2.980 to 6.801) | 3.731  (2.418 to 5.537) | −0.647  (−0.731 to −0.563) | < 0.001 |
| Somalia | 8.903  (5.830 to 13.088) | 7.999  (5.180 to 11.757) | −0.321  (−0.374 to −0.268) | < 0.001 |
| South Africa | 3.271  (2.147 to 4.647) | 3.427  (2.295 to 4.805) | 0.144  (0.090 to 0.199) | < 0.001 |
| South Sudan | 8.006  (5.147 to 11.796) | 5.649  (3.655 to 8.243) | −1.083  (−1.272 to −0.893) | < 0.001 |
| Spain | 20.361  (13.527 to 27.700) | 14.802  (10.028 to 19.943) | −1.038  (−1.105 to −0.971) | < 0.001 |
| Sri Lanka | 7.935  (5.427 to 11.172) | 5.683  (3.991 to 7.864) | −1.065  (−1.182 to −0.948) | < 0.001 |
| Sudan | 16.458  (10.799 to 24.007) | 12.093  (8.098 to 17.540) | −0.959  (−1.008 to −0.911) | < 0.001 |
| Suriname | 3.411  (2.253 to 4.900) | 3.026  (2.084 to 4.158) | −0.384  (−0.451 to −0.317) | < 0.001 |
| Sweden | 3.929  (2.703 to 5.400) | 3.685  (2.515 to 5.094) | −0.258  (−0.307 to −0.210) | < 0.001 |
| Switzerland | 8.207  (5.521 to 11.157) | 6.491  (4.415 to 8.841) | −0.766  (−0.795 to −0.737) | < 0.001 |
| Syrian Arab Republic | 18.250  (12.355 to 26.074) | 13.357  (8.937 to 18.619) | −1.006  (−1.051 to −0.960) | < 0.001 |
| Taiwan (Province of China) | 1.083  (0.693 to 1.644) | 1.068  (0.631 to 1.705) | −0.053  (−0.232 to 0.126) | 0.564 |
| Tajikistan | 5.256  (3.623 to 7.276) | 4.881  (3.307 to 6.897) | −0.229  (−0.279 to −0.179) | < 0.001 |
| Thailand | 7.766  (5.550 to 10.609) | 4.197  (2.717 to 6.102) | −1.946  (−2.026 to −1.866) | < 0.001 |
| Timor-Leste | 21.643  (14.973 to 30.667) | 14.809  (10.262 to 20.434) | −1.148  (−1.226 to −1.069) | < 0.001 |
| Togo | 9.500  (6.691 to 13.284) | 11.541  (8.010 to 16.148) | 0.627  (0.553 to 0.702) | < 0.001 |
| Tokelau | 4.520  (2.917 to 6.791) | 3.227  (2.077 to 4.766) | −1.078  (−1.182 to −0.974) | < 0.001 |
| Tonga | 4.277  (2.751 to 6.264) | 2.897  (1.859 to 4.142) | −1.223  (−1.319 to −1.126) | < 0.001 |
| Trinidad and Tobago | 1.770  (1.125 to 2.477) | 1.290  (0.853 to 1.827) | −1.006  (−1.085 to −0.927) | < 0.001 |
| Tunisia | 17.650  (11.411 to 25.352) | 11.669  (7.802 to 16.565) | −1.328  (−1.385 to −1.271) | < 0.001 |
| Turkey | 10.642  (7.214 to 15.290) | 7.996  (5.356 to 11.253) | −0.916  (−0.961 to −0.871) | < 0.001 |
| Turkmenistan | 3.758  (2.515 to 5.271) | 3.498  (2.343 to 5.038) | −0.208  (−0.281 to −0.136) | < 0.001 |
| Tuvalu | 5.069  (3.134 to 7.393) | 3.543  (2.265 to 5.274) | −1.147  (−1.225 to −1.069) | < 0.001 |
| Uganda | 6.840  (4.652 to 9.831) | 5.980  (4.141 to 8.466) | −0.370  (−0.456 to −0.284) | < 0.001 |
| Ukraine | 2.353  (1.528 to 3.324) | 2.094  (1.361 to 2.972) | −0.364  (−0.499 to −0.228) | < 0.001 |
| United Arab Emirates | 16.643  (11.108 to 23.607) | 11.636  (7.829 to 16.595) | −1.141  (−1.166 to −1.116) | < 0.001 |
| United Kingdom | 9.806  (6.646 to 13.182) | 7.607  (5.206 to 10.243) | −0.827  (−0.863 to −0.792) | < 0.001 |
| United Republic of Tanzania | 17.711  (12.399 to 24.443) | 12.735  (8.400 to 17.902) | −1.008  (−1.213 to −0.802) | < 0.001 |
| United States of America | 3.043  (2.050 to 4.213) | 2.604  (1.780 to 3.572) | −0.497  (−0.559 to −0.435) | < 0.001 |
| United States Virgin Islands | 1.744  (1.125 to 2.514) | 1.388  (0.902 to 1.963) | −0.708  (−0.767 to −0.649) | < 0.001 |
| Uruguay | 3.596  (2.312 to 5.084) | 2.983  (1.929 to 4.178) | −0.603  (−0.663 to −0.544) | < 0.001 |
| Uzbekistan | 5.167  (3.466 to 7.238) | 4.752  (3.168 to 6.690) | −0.244  (−0.315 to −0.173) | < 0.001 |
| Vanuatu | 4.520  (3.113 to 6.319) | 3.097  (2.021 to 4.610) | −1.196  (−1.253 to −1.14) | < 0.001 |
| Venezuela (Bolivarian Republic of) | 3.632  (2.396 to 5.068) | 3.251  (2.170 to 4.611) | −0.342  (−0.464 to −0.219) | < 0.001 |
| Viet Nam | 10.537  (7.100 to 15.199) | 7.347  (5.129 to 10.178) | −1.183  (−1.328 to −1.038) | < 0.001 |
| Yemen | 11.001  (7.419 to 15.718) | 14.111  (9.787 to 19.819) | 0.800  (0.745 to 0.855) | < 0.001 |
| Zambia | 7.586  (4.883 to 10.889) | 6.069  (4.027 to 8.584) | −0.889  (−1.177 to −0.601) | < 0.001 |
| Zimbabwe | 2.778  (1.846 to 3.944) | 2.508  (1.683 to 3.536) | −0.311  (−0.364 to −0.258) | < 0.001 |

ASYR = age-standardized years lived with disability rate per 100,000 populations; UI = uncertainty interval; AAPC = average annual percent change; CI = confidence interval

**Supplementary Table S4.** **National/regional burden of cataract in 1990 and 2021 with AAPC.**

| Country/Region | ASYR (95% UI) | | AAPC  (95% CI) | *P* value |
| --- | --- | --- | --- | --- |
|  | 1990 | 2021 |  |  |
| Afghanistan | 243.100  (173.933 to 323.094) | 196.312  (141.972 to 262.127) | −0.603  (−0.757 to −0.449) | < 0.001 |
| Albania | 24.050  (16.791 to 32.834) | 20.134  (14.042 to 27.807) | −0.573  (−0.601 to −0.544) | < 0.001 |
| Algeria | 156.670  (111.054 to 209.762) | 110.457  (77.671 to 148.495) | −1.121  (−1.136 to −1.106) | < 0.001 |
| American Samoa | 114.456  (83.448 to 153.249) | 88.676  (64.399 to 119.671) | −0.816  (−0.836 to −0.796) | < 0.001 |
| Andorra | 20.015  (13.862 to 27.430) | 18.674  (12.962 to 25.618) | −0.216  (−0.241 to −0.191) | < 0.001 |
| Angola | 80.418  (54.502 to 108.434) | 49.180  (34.113 to 66.985) | −1.572  (−1.608 to −1.536) | < 0.001 |
| Antigua and Barbuda | 44.107  (30.604 to 59.308) | 32.109  (22.753 to 43.426) | −1.024  (−1.059 to −0.988) | < 0.001 |
| Argentina | 35.705  (24.856 to 48.584) | 27.764  (19.316 to 37.628) | −0.808  (−0.829 to −0.787) | < 0.001 |
| Armenia | 72.774  (51.247 to 98.947) | 59.739  (41.111 to 82.305) | −0.638  (−0.655 to −0.620) | < 0.001 |
| Australia | 20.917  (14.718 to 28.084) | 19.386  (13.544 to 26.075) | −0.253  (−0.291 to −0.215) | < 0.001 |
| Austria | 21.104  (14.670 to 28.965) | 19.069  (13.286 to 26.246) | −0.326  (−0.355 to −0.296) | < 0.001 |
| Azerbaijan | 74.700  (52.395 to 101.097) | 62.150  (43.499 to 85.619) | −0.587  (−0.616 to −0.559) | < 0.001 |
| Bahamas | 41.411  (28.341 to 57.179) | 32.182  (22.368 to 43.710) | −0.813  (−0.831 to −0.795) | < 0.001 |
| Bahrain | 140.928  (100.186 to 190.708) | 95.790  (67.559 to 129.547) | −1.236  (−1.268 to −1.204) | < 0.001 |
| Bangladesh | 248.226  (179.690 to 330.164) | 167.165  (120.161 to 225.292) | −1.253  (−1.304 to −1.202) | < 0.001 |
| Barbados | 17.864  (12.592 to 24.050) | 15.172  (10.565 to 20.618) | −0.526  (−0.555 to −0.497) | < 0.001 |
| Belarus | 38.491  (26.532 to 52.884) | 31.847  (22.391 to 42.881) | −0.613  (−0.634 to −0.592) | < 0.001 |
| Belgium | 21.378  (15.022 to 29.186) | 19.213  (13.490 to 26.396) | −0.335  (−0.364 to −0.306) | < 0.001 |
| Belize | 54.910  (37.635 to 75.015) | 38.062  (26.730 to 51.843) | −1.179  (−1.226 to −1.133) | < 0.001 |
| Benin | 99.315  (69.765 to 134.065) | 126.058  (90.616 to 168.690) | 0.769  (0.720 to 0.819) | < 0.001 |
| Bermuda | 38.680  (26.531 to 52.470) | 29.213  (20.683 to 39.827) | −0.906  (−0.917 to −0.894) | < 0.001 |
| Bhutan | 90.044  (61.806 to 123.341) | 48.781  (34.512 to 65.199) | −1.962  (−2.012 to −1.912) | < 0.001 |
| Bolivia (Plurinational State of) | 144.084  (100.452 to 194.665) | 98.592  (69.388 to 134.181) | −1.216  (−1.239 to −1.193) | < 0.001 |
| Bosnia and Herzegovina | 23.545  (16.579 to 31.964) | 18.833  (13.029 to 26.107) | −0.717  (−0.755 to −0.678) | < 0.001 |
| Botswana | 99.410  (68.712 to 135.357) | 68.907  (48.968 to 92.815) | −1.177  (−1.198 to −1.156) | < 0.001 |
| Brazil | 99.024  (70.349 to 131.486) | 68.693  (48.927 to 91.879) | −1.179  (−1.267 to −1.09) | < 0.001 |
| Brunei Darussalam | 22.037  (15.460 to 30.346) | 18.486  (12.997 to 25.547) | −0.546  (−0.598 to −0.494) | < 0.001 |
| Bulgaria | 11.649  (8.240 to 15.982) | 11.865  (7.975 to 16.401) | 0.080  (0.026 to 0.135) | 0.004 |
| Burkina Faso | 72.901  (50.284 to 99.263) | 104.518  (74.672 to 141.525) | 1.164  (1.088 to 1.240) | < 0.001 |
| Burundi | 38.118  (26.572 to 50.976) | 31.664  (22.379 to 42.474) | −0.698  (−0.748 to −0.648) | < 0.001 |
| Cabo Verde | 125.051  (90.262 to 169.248) | 83.184  (58.902 to 111.670) | −1.310  (−1.327 to −1.294) | < 0.001 |
| Cambodia | 341.485  (248.319 to 457.901) | 199.265  (144.708 to 266.668) | −1.727  (−1.777 to −1.676) | < 0.001 |
| Cameroon | 86.866  (61.352 to 119.239) | 70.765  (49.999 to 94.582) | −0.651  (−0.696 to −0.607) | < 0.001 |
| Canada | 17.338  (12.247 to 23.468) | 16.252  (11.553 to 22.204) | −0.195  (−0.234 to −0.156) | < 0.001 |
| Central African Republic | 36.591  (25.010 to 49.506) | 39.171  (26.907 to 53.236) | 0.223  (0.198 to 0.249) | < 0.001 |
| Chad | 116.010  (81.145 to 155.994) | 112.076  (80.084 to 151.909) | −0.113  (−0.148 to −0.077) | < 0.001 |
| Chile | 55.206  (38.724 to 74.506) | 40.573  (28.920 to 55.788) | −0.991  (−1.021 to −0.962) | < 0.001 |
| China | 66.970  (47.899 to 89.472) | 55.893  (40.173 to 75.513) | −0.442  (−0.699 to −0.184) | 0.001 |
| Colombia | 84.658  (58.378 to 114.340) | 56.664  (39.670 to 77.590) | −1.290  (−1.320 to −1.260) | < 0.001 |
| Comoros | 118.314  (82.476 to 159.375) | 82.642  (57.634 to 112.645) | −1.146  (−1.176 to −1.116) | < 0.001 |
| Congo | 57.728  (39.885 to 77.729) | 40.056  (27.694 to 54.627) | −1.173  (−1.203 to −1.143) | < 0.001 |
| Cook Islands | 153.788  (111.666 to 205.360) | 102.446  (73.544 to 138.249) | −1.298  (−1.335 to −1.261) | < 0.001 |
| Costa Rica | 83.866  (57.978 to 112.982) | 63.111  (45.124 to 86.554) | −0.918  (−0.959 to −0.878) | < 0.001 |
| Croatia | 20.191  (14.147 to 27.859) | 17.978  (12.724 to 25.055) | −0.368  (−0.391 to −0.346) | < 0.001 |
| Cuba | 69.007  (47.617 to 94.367) | 50.739  (35.195 to 69.441) | −0.987  (−1.013 to −0.961) | < 0.001 |
| Cyprus | 21.542  (15.107 to 29.696) | 19.096  (13.363 to 26.044) | −0.374  (−0.420 to −0.329) | < 0.001 |
| Czechia | 19.966  (13.961 to 27.132) | 17.398  (11.997 to 24.232) | −0.436  (−0.474 to −0.398) | < 0.001 |
| Democratic People's Republic of Korea | 23.771  (16.664 to 33.392) | 18.006  (12.006 to 26.927) | −0.910  (−0.97 to −0.851) | < 0.001 |
| Democratic Republic of the Congo | 15.653  (10.966 to 21.729) | 17.329  (12.032 to 24.004) | 0.338  (0.293 to 0.383) | < 0.001 |
| Denmark | 22.154  (15.150 to 30.659) | 20.627  (14.051 to 29.516) | −0.215  (−0.249 to −0.182) | < 0.001 |
| Djibouti | 119.397  (83.711 to 161.741) | 83.583  (59.539 to 112.780) | −1.141  (−1.177 to −1.106) | < 0.001 |
| Dominica | 47.940  (33.242 to 65.499) | 34.236  (23.671 to 47.124) | −1.081  (−1.102 to −1.060) | < 0.001 |
| Dominican Republic | 52.821  (35.826 to 72.342) | 37.753  (26.142 to 51.704) | −1.075  (−1.099 to −1.051) | < 0.001 |
| Ecuador | 84.169  (59.288 to 114.586) | 64.108  (45.762 to 88.439) | −0.867  (−0.899 to −0.836) | < 0.001 |
| Egypt | 137.263  (95.840 to 186.074) | 118.430  (83.207 to 159.747) | −0.469  (−0.557 to −0.381) | < 0.001 |
| El Salvador | 95.095  (64.437 to 130.214) | 56.292  (38.786 to 76.747) | −1.682  (−1.715 to −1.648) | < 0.001 |
| Equatorial Guinea | 311.615  (213.898 to 422.449) | 108.174  (74.994 to 145.479) | −3.366  (−3.421 to −3.310) | < 0.001 |
| Eritrea | 138.051  (96.652 to 184.575) | 93.967  (65.913 to 127.915) | −1.225  (−1.284 to −1.165) | < 0.001 |
| Estonia | 26.147  (18.062 to 35.551) | 21.503  (14.712 to 29.631) | −0.633  (−0.663 to −0.603) | < 0.001 |
| Eswatini | 124.032  (86.242 to 169.297) | 85.504  (59.859 to 114.196) | −1.190  (−1.218 to −1.162) | < 0.001 |
| Ethiopia | 247.033  (177.348 to 330.749) | 222.529  (158.172 to 294.173) | −0.334  (−0.352 to −0.316) | < 0.001 |
| Fiji | 165.124  (120.367 to 220.163) | 124.282  (89.588 to 168.304) | −0.927  (−1.044 to −0.810) | < 0.001 |
| Finland | 19.233  (13.543 to 26.013) | 16.874  (11.877 to 23.407) | −0.420  (−0.473 to −0.367) | < 0.001 |
| France | 21.847  (15.473 to 30.203) | 19.912  (13.852 to 27.568) | −0.292  (−0.317 to −0.267) | < 0.001 |
| Gabon | 59.047  (40.322 to 80.056) | 37.210  (25.170 to 50.464) | −1.475  (−1.495 to −1.455) | < 0.001 |
| Gambia | 182.174  (131.158 to 243.193) | 144.068  (103.636 to 194.059) | −0.761  (−0.792 to −0.730) | < 0.001 |
| Georgia | 67.612  (47.695 to 92.653) | 60.576  (42.494 to 82.791) | −0.350  (−0.391 to −0.31) | < 0.001 |
| Germany | 20.154  (14.123 to 27.767) | 18.389  (12.745 to 25.612) | −0.293  (−0.322 to −0.264) | < 0.001 |
| Ghana | 76.921  (54.249 to 105.068) | 85.188  (60.818 to 116.839) | 0.317  (0.229 to 0.404) | < 0.001 |
| Greece | 23.398  (16.446 to 31.998) | 20.742  (14.407 to 28.948) | −0.381  (−0.426 to −0.336) | < 0.001 |
| Greenland | 19.966  (13.983 to 27.209) | 18.150  (12.535 to 24.639) | −0.307  (−0.363 to −0.251) | < 0.001 |
| Grenada | 53.953  (37.367 to 72.994) | 35.496  (24.676 to 47.992) | −1.341  (−1.376 to −1.306) | < 0.001 |
| Guam | 109.031  (77.893 to 147.152) | 90.594  (65.572 to 123.973) | −0.594  (−0.634 to −0.555) | < 0.001 |
| Guatemala | 102.697  (71.470 to 140.237) | 68.473  (47.516 to 93.895) | −1.299  (−1.327 to −1.270) | < 0.001 |
| Guinea | 121.322  (84.327 to 161.780) | 114.998  (81.988 to 154.662) | −0.180  (−0.218 to −0.141) | < 0.001 |
| Guinea-Bissau | 77.496  (53.064 to 105.331) | 74.806  (52.346 to 102.042) | −0.117  (−0.237 to 0.002) | 0.054 |
| Guyana | 57.384  (40.152 to 77.882) | 37.914  (26.561 to 52.195) | −1.332  (−1.366 to −1.298) | < 0.001 |
| Haiti | 78.616  (54.074 to 105.483) | 53.061  (36.771 to 71.944) | −1.268  (−1.300 to −1.236) | < 0.001 |
| Honduras | 63.015  (42.849 to 85.579) | 39.966  (27.698 to 54.964) | −1.464  (−1.507 to −1.420) | < 0.001 |
| Hungary | 34.555  (24.584 to 46.812) | 29.009  (20.702 to 40.085) | −0.562  (−0.580 to −0.544) | < 0.001 |
| Iceland | 19.279  (12.859 to 26.785) | 19.255  (12.808 to 27.450) | −0.015  (−0.086 to 0.057) | 0.685 |
| India | 283.047  (204.741 to 375.913) | 187.820  (137.594 to 248.671) | −1.293  (−1.325 to −1.262) | < 0.001 |
| Indonesia | 230.301  (167.651 to 306.434) | 163.216  (119.157 to 217.718) | −1.108  (−1.141 to −1.074) | < 0.001 |
| Iran (Islamic Republic of) | 109.563  (77.945 to 145.962) | 90.753  (65.205 to 121.800) | −0.590  (−0.652 to −0.528) | < 0.001 |
| Iraq | 166.796  (115.626 to 223.443) | 109.993  (78.941 to 148.684) | −1.337  (−1.363 to −1.311) | < 0.001 |
| Ireland | 21.421  (14.961 to 29.193) | 19.133  (13.255 to 25.918) | −0.356  (−0.412 to −0.301) | < 0.001 |
| Israel | 21.368  (14.921 to 29.516) | 19.684  (13.910 to 27.400) | −0.25  (−0.288 to −0.213) | < 0.001 |
| Italy | 47.406  (34.226 to 64.826) | 39.618  (28.404 to 54.133) | −0.581  (−0.612 to −0.55) | < 0.001 |
| Ivory Coast | 57.588  (40.257 to 78.457) | 103.058  (73.239 to 138.631) | 1.893  (1.708 to 2.078) | < 0.001 |
| Jamaica | 46.182  (31.869 to 63.226) | 34.204  (23.812 to 46.533) | −0.968  (−0.988 to −0.948) | < 0.001 |
| Japan | 18.334  (13.07 to 24.7) | 16.642  (11.828 to 22.553) | −0.301  (−0.372 to −0.229) | < 0.001 |
| Jordan | 66.400  (45.736 to 89.676) | 47.812  (33.196 to 64.970) | −1.042  (−1.12 to −0.964) | < 0.001 |
| Kazakhstan | 71.226  (49.650 to 96.746) | 59.577  (42.020 to 81.270) | −0.571  (−0.586 to −0.557) | < 0.001 |
| Kenya | 188.687  (135.895 to 254.639) | 127.669  (92.471 to 170.626) | −1.249  (−1.298 to −1.201) | < 0.001 |
| Kiribati | 150.646  (109.471 to 201.448) | 118.797  (85.825 to 158.829) | −0.757  (−0.803 to −0.711) | < 0.001 |
| Kuwait | 118.701  (84.724 to 159.767) | 89.999  (63.557 to 122.779) | −0.891  (−0.919 to −0.864) | < 0.001 |
| Kyrgyzstan | 73.009  (50.825 to 98.230) | 61.479  (43.886 to 82.853) | −0.553  (−0.583 to −0.524) | < 0.001 |
| Lao People's Democratic Republic | 52.585  (36.856 to 71.228) | 40.897  (28.733 to 56.819) | −0.808  (−0.837 to −0.78) | < 0.001 |
| Latvia | 36.708  (25.862 to 49.833) | 31.126  (21.741 to 42.634) | −0.537  (−0.564 to −0.51) | < 0.001 |
| Lebanon | 178.616  (126.984 to 236.804) | 111.219  (79.818 to 151.231) | −1.517  (−1.536 to −1.499) | < 0.001 |
| Lesotho | 138.647  (96.563 to 188.139) | 98.161  (68.774 to 131.911) | −1.105  (−1.136 to −1.074) | < 0.001 |
| Liberia | 130.130  (92.634 to 173.601) | 94.253  (66.809 to 125.941) | −1.042  (−1.068 to −1.017) | < 0.001 |
| Libya | 180.752  (127.020 to 244.714) | 120.489  (85.693 to 161.309) | −1.296  (−1.323 to −1.269) | < 0.001 |
| Lithuania | 36.616  (25.595 to 49.101) | 30.615  (21.666 to 41.936) | −0.577  (−0.600 to −0.553) | < 0.001 |
| Luxembourg | 20.969  (14.343 to 28.917) | 18.811  (13.148 to 25.433) | −0.344  (−0.389 to −0.299) | < 0.001 |
| Madagascar | 60.376  (41.551 to 82.597) | 45.974  (31.989 to 62.754) | −0.855  (−0.940 to −0.770) | < 0.001 |
| Malawi | 121.425  (86.611 to 160.989) | 78.814  (55.716 to 105.842) | −1.398  (−1.423 to −1.372) | < 0.001 |
| Malaysia | 210.724  (151.942 to 282.725) | 133.057  (95.525 to 179.057) | −1.478  (−1.508 to −1.448) | < 0.001 |
| Maldives | 69.288  (47.307 to 93.739) | 38.298  (26.405 to 52.826) | −1.905  (−1.978 to −1.832) | < 0.001 |
| Mali | 177.029  (125.309 to 235.812) | 161.617  (117.288 to 218.047) | −0.299  (−0.346 to −0.252) | < 0.001 |
| Malta | 21.764  (15.120 to 29.892) | 19.360  (13.358 to 26.758) | −0.361  (−0.392 to −0.330) | < 0.001 |
| Marshall Islands | 147.585  (106.463 to 196.824) | 103.280  (73.991 to 139.823) | −1.140  (−1.166 to −1.115) | < 0.001 |
| Mauritania | 126.255  (90.397 to 169.719) | 94.938  (67.148 to 129.813) | −0.920  (−0.935 to −0.905) | < 0.001 |
| Mauritius | 127.558  (90.874 to 172.890) | 91.205  (64.180 to 123.356) | −1.075  (−1.091 to −1.058) | < 0.001 |
| Mexico | 98.750  (70.849 to 132.401) | 69.409  (49.496 to 93.938) | −1.155  (−1.211 to −1.098) | < 0.001 |
| Micronesia (Federated States of) | 147.953  (107.003 to 198.275) | 108.909  (78.291 to 146.946) | −0.978  (−1.013 to −0.942) | < 0.001 |
| Monaco | 20.381  (14.156 to 28.165) | 19.016  (13.129 to 26.622) | −0.216  (−0.237 to −0.195) | < 0.001 |
| Mongolia | 60.742  (41.427 to 82.551) | 43.174  (29.331 to 59.206) | −1.090  (−1.122 to −1.058) | < 0.001 |
| Montenegro | 19.922  (13.907 to 27.723) | 18.266  (12.586 to 25.287) | −0.281  (−0.322 to −0.240) | < 0.001 |
| Morocco | 135.193  (98.749 to 179.455) | 101.332  (72.887 to 136.082) | −0.926  (−0.94 to −0.911) | < 0.001 |
| Mozambique | 122.862  (85.403 to 165.003) | 95.666  (68.423 to 126.384) | −0.824  (−0.877 to −0.771) | < 0.001 |
| Myanmar | 302.335  (214.444 to 409.094) | 194.690  (139.335 to 263.934) | −1.418  (−1.459 to −1.378) | < 0.001 |
| Namibia | 116.351  (81.669 to 158.326) | 79.172  (55.732 to 106.192) | −1.238  (−1.253 to −1.223) | < 0.001 |
| Nauru | 131.247  (95.481 to 176.129) | 105.434  (75.976 to 141.176) | −0.698  (−0.749 to −0.647) | < 0.001 |
| Nepal | 127.748  (91.002 to 171.076) | 95.755  (67.926 to 130.623) | −0.937  (−1.007 to −0.868) | < 0.001 |
| Netherlands | 16.089  (11.118 to 21.953) | 14.910  (10.195 to 20.733) | −0.245  (−0.288 to −0.202) | < 0.001 |
| New Zealand | 24.600  (17.738 to 33.108) | 20.938  (15.091 to 28.495) | −0.518  (−0.549 to −0.487) | < 0.001 |
| Nicaragua | 102.506  (70.784 to 139.498) | 64.532  (44.938 to 87.827) | −1.482  (−1.501 to −1.462) | < 0.001 |
| Niger | 129.111  (90.053 to 172.524) | 135.401  (96.141 to 184.018) | 0.161  (0.118 to 0.205) | < 0.001 |
| Nigeria | 237.751  (173.548 to 314.937) | 206.560  (150.761 to 277.627) | −0.458  (−0.473 to −0.443) | < 0.001 |
| Niue | 127.171  (92.303 to 169.674) | 92.749  (66.574 to 125.876) | −1.010  (−1.033 to −0.987) | < 0.001 |
| North Macedonia | 22.039  (15.175 to 30.290) | 18.680  (12.918 to 25.559) | −0.532  (−0.567 to −0.496) | < 0.001 |
| Northern Mariana Islands | 105.821  (75.836 to 142.972) | 88.946  (63.765 to 122.495) | −0.553  (−0.587 to −0.519) | < 0.001 |
| Norway | 17.962  (12.697 to 24.907) | 16.991  (11.940 to 23.503) | −0.152  (−0.242 to −0.063) | 0.001 |
| Oman | 235.997  (162.228 to 316.696) | 186.598  (133.409 to 251.817) | −0.760  (−0.786 to −0.734) | < 0.001 |
| Pakistan | 344.364  (250.538 to 452.214) | 308.437  (226.006 to 406.808) | −0.382  (−0.437 to −0.326) | < 0.001 |
| Palau | 118.014  (84.725 to 158.209) | 90.270  (64.995 to 122.371) | −0.861  (−0.877 to −0.845) | < 0.001 |
| Palestine | 206.359  (147.806 to 274.336) | 130.052  (93.453 to 174.313) | −1.471  (−1.517 to −1.426) | < 0.001 |
| Panama | 132.946  (92.599 to 179.603) | 93.916  (67.275 to 125.116) | −1.124  (−1.153 to −1.094) | < 0.001 |
| Papua New Guinea | 224.758  (164.655 to 299.001) | 185.669  (133.837 to 247.706) | −0.620  (−0.666 to −0.574) | < 0.001 |
| Paraguay | 118.940  (85.074 to 155.860) | 64.998  (45.326 to 87.249) | −1.925  (−1.974 to −1.876) | < 0.001 |
| Peru | 170.139  (119.825 to 229.078) | 104.296  (73.536 to 141.428) | −1.572  (−1.623 to −1.521) | < 0.001 |
| Philippines | 149.397  (108.626 to 199.389) | 122.949  (88.991 to 165.804) | −0.631  (−0.653 to −0.609) | < 0.001 |
| Poland | 21.119  (14.921 to 28.827) | 19.135  (13.551 to 26.475) | −0.315  (−0.324 to −0.305) | < 0.001 |
| Portugal | 22.943  (16.006 to 31.015) | 19.763  (13.588 to 27.441) | −0.467  (−0.505 to −0.429) | < 0.001 |
| Puerto Rico | 38.731  (27.170 to 52.835) | 28.710  (20.183 to 39.277) | −0.961  (−0.984 to −0.939) | < 0.001 |
| Qatar | 139.669  (100.155 to 188.221) | 92.088  (65.495 to 125.015) | −1.332  (−1.431 to −1.232) | < 0.001 |
| Republic of Korea | 26.092  (18.102 to 34.801) | 18.604  (13.114 to 25.483) | −1.077  (−1.15 to −1.005) | < 0.001 |
| Republic of Moldova | 70.268  (49.527 to 96.008) | 60.114  (42.299 to 82.131) | −0.507  (−0.538 to −0.476) | < 0.001 |
| Romania | 22.323  (15.611 to 30.470) | 19.351  (13.342 to 26.890) | −0.459  (−0.498 to −0.420) | < 0.001 |
| Russian Federation | 32.267  (22.877 to 43.674) | 26.397  (18.611 to 35.935) | −0.645  (−0.666 to −0.623) | < 0.001 |
| Rwanda | 48.100  (32.890 to 65.304) | 30.813  (20.932 to 41.945) | −1.426  (−1.462 to −1.390) | < 0.001 |
| Saint Kitts and Nevis | 48.820  (33.593 to 66.490) | 33.247  (23.516 to 45.572) | −1.235  (−1.266 to −1.203) | < 0.001 |
| Saint Lucia | 49.859  (34.351 to 68.127) | 33.594  (23.626 to 45.925) | −1.269  (−1.302 to −1.235) | < 0.001 |
| Saint Vincent and the Grenadines | 52.795  (36.306 to 71.652) | 36.422  (25.399 to 49.386) | −1.192  (−1.216 to −1.169) | < 0.001 |
| Samoa | 131.428  (95.283 to 175.356) | 100.834  (72.794 to 134.898) | −0.846  (−0.877 to −0.815) | < 0.001 |
| San Marino | 20.252  (14.021 to 28.173) | 18.932  (13.190 to 26.159) | −0.214  (−0.236 to −0.191) | < 0.001 |
| Sao Tome and Principe | 137.194  (96.772 to 186.428) | 96.459  (69.060 to 131.717) | −1.132  (−1.154 to −1.11) | < 0.001 |
| Saudi Arabia | 381.522  (275.076 to 505.326) | 182.845  (132.607 to 245.365) | −2.342  (−2.364 to −2.321) | < 0.001 |
| Senegal | 100.870  (70.563 to 134.183) | 74.144  (51.522 to 100.781) | −0.955  (−1.030 to −0.879) | < 0.001 |
| Serbia | 21.613  (15.009 to 29.769) | 18.583  (12.824 to 25.986) | −0.490  (−0.519 to −0.462) | < 0.001 |
| Seychelles | 129.804  (92.463 to 176.685) | 93.414  (66.810 to 127.164) | −1.052  (−1.067 to −1.037) | < 0.001 |
| Sierra Leone | 75.232  (49.658 to 104.257) | 57.838  (39.506 to 78.412) | −0.845  (−0.897 to −0.793) | < 0.001 |
| Singapore | 26.917  (18.941 to 36.855) | 22.804  (15.854 to 31.128) | −0.523  (−0.574 to −0.472) | < 0.001 |
| Slovakia | 20.692  (14.349 to 28.403) | 18.495  (12.843 to 25.441) | −0.356  (−0.385 to −0.326) | < 0.001 |
| Slovenia | 19.425  (13.636 to 27.034) | 17.532  (12.556 to 24.41) | −0.331  (−0.357 to −0.304) | < 0.001 |
| Solomon Islands | 161.194  (116.387 to 216.206) | 124.861  (90.966 to 167.955) | −0.811  (−0.83 to −0.793) | < 0.001 |
| Somalia | 99.134  (67.566 to 134.943) | 89.198  (63.140 to 119.818) | −0.335  (−0.366 to −0.305) | < 0.001 |
| South Africa | 117.234  (82.609 to 156.813) | 81.996  (57.851 to 108.631) | −1.143  (−1.216 to −1.07) | < 0.001 |
| South Sudan | 272.041  (192.317 to 363.020) | 196.786  (140.338 to 262.480) | −1.034  (−1.166 to −0.902) | < 0.001 |
| Spain | 47.074  (32.846 to 64.836) | 43.201  (30.382 to 59.517) | −0.264  (−0.293 to −0.236) | < 0.001 |
| Sri Lanka | 131.193  (92.522 to 177.652) | 99.619  (71.169 to 136.237) | −0.879  (−0.905 to −0.854) | < 0.001 |
| Sudan | 151.280  (105.776 to 203.414) | 87.085  (61.447 to 117.776) | −1.722  (−1.802 to −1.641) | < 0.001 |
| Suriname | 82.245  (56.935 to 110.891) | 60.333  (42.822 to 79.805) | −0.995  (−1.011 to −0.978) | < 0.001 |
| Sweden | 12.798  (8.980 to 17.849) | 12.592  (8.797 to 17.469) | −0.053  (−0.105 to −0.001) | 0.046 |
| Switzerland | 19.735  (13.709 to 27.204) | 18.397  (12.744 to 25.596) | −0.209  (−0.250 to −0.168) | < 0.001 |
| Syrian Arab Republic | 175.897  (124.658 to 235.603) | 112.698  (79.939 to 150.400) | −1.425  (−1.464 to −1.386) | < 0.001 |
| Taiwan (Province of China) | 9.625  (6.208 to 14.864) | 9.640  (5.843 to 14.671) | −0.001  (−0.071 to 0.068) | 0.967 |
| Tajikistan | 80.778  (56.468 to 110.399) | 69.437  (49.274 to 94.182) | −0.489  (−0.510 to −0.467) | < 0.001 |
| Thailand | 142.951  (103.781 to 194.181) | 98.496  (68.244 to 136.799) | −1.189  (−1.230 to −1.149) | < 0.001 |
| Timor-Leste | 176.047  (123.152 to 236.915) | 101.996  (71.920 to 135.673) | −1.671  (−1.828 to −1.514) | < 0.001 |
| Togo | 105.642  (74.725 to 141.316) | 105.575  (74.869 to 141.982) | −0.010  (−0.048 to 0.028) | 0.606 |
| Tokelau | 142.915  (103.390 to 191.127) | 99.579  (71.938 to 133.836) | −1.151  (−1.185 to −1.117) | < 0.001 |
| Tonga | 80.456  (58.471 to 107.573) | 59.675  (42.788 to 80.234) | −0.96  (−0.997 to −0.923) | < 0.001 |
| Trinidad and Tobago | 42.071  (28.752 to 57.802) | 29.086  (20.348 to 39.341) | −1.194  (−1.232 to −1.156) | < 0.001 |
| Tunisia | 153.071  (108.594 to 206.157) | 91.001  (64.867 to 121.595) | −1.662  (−1.683 to −1.641) | < 0.001 |
| Turkey | 96.878  (69.021 to 129.918) | 65.407  (46.550 to 89.765) | −1.261  (−1.307 to −1.216) | < 0.001 |
| Turkmenistan | 142.802  (103.211 to 196.957) | 110.331  (78.225 to 152.618) | −0.830  (−0.852 to −0.808) | < 0.001 |
| Tuvalu | 156.701  (113.161 to 209.013) | 108.120  (77.837 to 145.905) | −1.182  (−1.222 to −1.142) | < 0.001 |
| Uganda | 56.436  (41.008 to 76.317) | 50.484  (35.401 to 68.446) | −0.328  (−0.390 to −0.265) | < 0.001 |
| Ukraine | 39.027  (27.621 to 52.361) | 34.951  (24.846 to 47.322) | −0.357  (−0.381 to −0.334) | < 0.001 |
| United Arab Emirates | 134.421  (94.921 to 180.949) | 88.176  (62.534 to 119.959) | −1.345  (−1.377 to −1.313) | < 0.001 |
| United Kingdom | 21.981  (15.531 to 30.401) | 19.574  (13.721 to 27.041) | −0.366  (−0.377 to −0.355) | < 0.001 |
| United Republic of Tanzania | 116.480  (82.915 to 155.969) | 94.192  (66.836 to 127.304) | −0.692  (−0.747 to −0.637) | < 0.001 |
| United States of America | 17.895  (12.774 to 23.982) | 16.656  (11.849 to 22.290) | −0.202  (−0.262 to −0.142) | < 0.001 |
| United States Virgin Islands | 40.826  (28.469 to 55.427) | 30.950  (21.905 to 42.010) | −0.890  (−0.916 to −0.864) | < 0.001 |
| Uruguay | 14.425  (10.010 to 20.108) | 12.464  (8.583 to 17.275) | −0.471  (−0.500 to −0.442) | < 0.001 |
| Uzbekistan | 77.857  (53.701 to 106.004) | 64.181  (45.155 to 87.121) | −0.621  (−0.642 to −0.600) | < 0.001 |
| Vanuatu | 103.955  (74.884 to 138.880) | 87.014  (63.026 to 118.352) | −0.566  (−0.608 to −0.524) | < 0.001 |
| Venezuela (Bolivarian Republic of) | 75.226  (51.394 to 102.491) | 49.734  (34.132 to 67.777) | −1.307  (−1.380 to −1.234) | < 0.001 |
| Viet Nam | 126.091  (89.695 to 168.163) | 89.289  (64.851 to 121.106) | −1.132  (−1.210 to −1.053) | < 0.001 |
| Yemen | 124.344  (87.934 to 166.818) | 120.337  (85.005 to 161.042) | −0.120  (−0.174 to −0.066) | < 0.001 |
| Zambia | 102.496  (72.247 to 137.636) | 75.956  (53.524 to 102.198) | −0.954  (−0.988 to −0.920) | < 0.001 |
| Zimbabwe | 130.973  (92.214 to 173.744) | 98.075  (68.458 to 131.280) | −0.924  (−0.949 to −0.899) | < 0.001 |

ASYR = age-standardized years lived with disability rate per 100,000 populations; UI = uncertainty interval; AAPC = average annual percent change; CI = confidence interval

**Supplementary Table S5. National/regional burden of glaucoma in 1990 and 2021 with AAPC.**

| Country/Region | ASYR (95% UI) | | AAPC  (95% CI) | *P* value |
| --- | --- | --- | --- | --- |
|  | 1990 | 2021 |  |  |
| Afghanistan | 46.447  (30.477 to 66.513) | 34.185  (22.453 to 48.171) | −0.909  (−1.029 to −0.789) | < 0.001 |
| Albania | 6.408  (4.318 to 9.204) | 4.296  (2.868 to 5.997) | −1.287  (−1.344 to −1.23) | < 0.001 |
| Algeria | 39.086  (26.520 to 55.034) | 23.474  (15.784 to 33.039) | −1.625  (−1.666 to −1.584) | < 0.001 |
| American Samoa | 11.298  (7.367 to 16.204) | 8.014  (5.387 to 11.147) | −1.139  (−1.271 to −1.008) | < 0.001 |
| Andorra | 6.737  (4.402 to 9.447) | 5.209  (3.516 to 7.461) | −0.838  (−0.86 to −0.816) | < 0.001 |
| Angola | 32.630  (21.900 to 46.605) | 21.122  (14.261 to 30.066) | −1.399  (−1.446 to −1.353) | < 0.001 |
| Antigua and Barbuda | 15.804  (10.615 to 22.179) | 10.315  (6.929 to 14.356) | −1.365  (−1.407 to −1.323) | < 0.001 |
| Argentina | 8.812  (5.888 to 12.473) | 5.997  (4.063 to 8.281) | −1.229  (−1.295 to −1.163) | < 0.001 |
| Armenia | 15.183  (10.226 to 21.535) | 10.220  (6.901 to 14.407) | −1.281  (−1.336 to −1.226) | < 0.001 |
| Australia | 6.051  (4.159 to 8.335) | 4.841  (3.260 to 6.725) | −0.717  (−0.771 to −0.663) | < 0.001 |
| Austria | 7.625  (5.189 to 10.892) | 5.449  (3.677 to 7.679) | −1.085  (−1.131 to −1.038) | < 0.001 |
| Azerbaijan | 15.817  (10.406 to 22.757) | 11.327  (7.597 to 15.926) | −1.077  (−1.143 to −1.012) | < 0.001 |
| Bahamas | 15.106  (10.231 to 21.547) | 10.631  (7.093 to 15.172) | −1.134  (−1.198 to −1.070) | < 0.001 |
| Bahrain | 34.000  (22.609 to 48.126) | 18.377  (12.468 to 25.374) | −1.965  (−2.001 to −1.928) | < 0.001 |
| Bangladesh | 14.923  (9.820 to 20.773) | 7.983  (5.452 to 11.307) | −2.011  (−2.082 to −1.939) | < 0.001 |
| Barbados | 6.695  (4.590 to 9.240) | 4.816  (3.268 to 6.642) | −1.060  (−1.124 to −0.996) | < 0.001 |
| Belarus | 7.316  (4.955 to 10.499) | 5.018  (3.374 to 7.013) | −1.202  (−1.288 to −1.116) | < 0.001 |
| Belgium | 7.835  (5.289 to 11.052) | 5.615  (3.714 to 7.948) | −1.070  (−1.134 to −1.005) | < 0.001 |
| Belize | 20.529  (13.476 to 29.181) | 13.505  (8.840 to 19.253) | −1.334  (−1.368 to −1.301) | < 0.001 |
| Benin | 29.224  (19.510 to 40.657) | 32.124  (21.402 to 43.931) | 0.315  (0.199 to 0.430) | < 0.001 |
| Bermuda | 13.238  (8.816 to 18.717) | 8.319  (5.746 to 11.681) | −1.494  (−1.518 to −1.471) | < 0.001 |
| Bhutan | 12.083  (7.938 to 17.120) | 6.118  (4.158 to 8.307) | −2.183  (−2.285 to −2.081) | < 0.001 |
| Bolivia (Plurinational State of) | 25.183  (16.494 to 35.542) | 15.747  (10.499 to 22.16) | −1.500  (−1.552 to −1.449) | < 0.001 |
| Bosnia and Herzegovina | 6.202  (4.138 to 8.834) | 3.767  (2.545 to 5.264) | −1.611  (−1.722 to −1.500) | < 0.001 |
| Botswana | 62.240  (42.297 to 89.426) | 38.311  (25.901 to 54.372) | −1.555  (−1.601 to −1.510) | < 0.001 |
| Brazil | 22.400  (15.513 to 30.492) | 15.758  (10.868 to 21.188) | −1.109  (−1.163 to −1.055) | < 0.001 |
| Brunei Darussalam | 11.124  (7.557 to 15.484) | 7.507  (5.139 to 10.493) | −1.258  (−1.296 to −1.220) | < 0.001 |
| Bulgaria | 3.646  (2.495 to 5.084) | 2.672  (1.787 to 3.741) | −0.995  (−1.048 to −0.943) | < 0.001 |
| Burkina Faso | 25.596  (16.671 to 36.842) | 30.156  (19.920 to 43.530) | 0.497  (0.324 to 0.670) | < 0.001 |
| Burundi | 18.803  (13.199 to 25.848) | 17.419  (11.997 to 24.112) | −0.275  (−0.301 to −0.250) | < 0.001 |
| Cabo Verde | 24.464  (16.347 to 33.635) | 18.387  (12.291 to 25.603) | −0.926  (−0.992 to −0.860) | < 0.001 |
| Cambodia | 13.546  (9.040 to 19.378) | 7.792  (5.113 to 11.017) | −1.743  (−1.788 to −1.699) | < 0.001 |
| Cameroon | 31.984  (21.486 to 44.391) | 24.978  (17.196 to 35.128) | −0.786  (−0.824 to −0.747) | < 0.001 |
| Canada | 4.897  (3.340 to 6.954) | 4.380  (2.999 to 6.061) | −0.365  (−0.393 to −0.336) | < 0.001 |
| Central African Republic | 15.912  (10.682 to 22.217) | 16.354  (11.038 to 22.878) | 0.090  (0.046 to 0.134) | < 0.001 |
| Chad | 37.400  (24.266 to 52.617) | 35.529  (23.661 to 49.604) | −0.168  (−0.210 to −0.125) | < 0.001 |
| Chile | 8.573  (5.685 to 12.292) | 5.752  (3.904 to 8.048) | −1.274  (−1.315 to −1.234) | < 0.001 |
| China | 10.490  (7.189 to 14.534) | 4.913  (3.348 to 6.853) | −2.460  (−2.634 to −2.286) | < 0.001 |
| Colombia | 17.838  (11.838 to 25.347) | 10.944  (7.402 to 15.120) | −1.563  (−1.597 to −1.529) | < 0.001 |
| Comoros | 36.579  (24.477 to 50.462) | 25.466  (17.209 to 35.975) | −1.158  (−1.182 to −1.134) | < 0.001 |
| Congo | 23.206  (15.996 to 32.403) | 16.539  (11.467 to 22.662) | −1.091  (−1.135 to −1.047) | < 0.001 |
| Cook Islands | 20.614  (13.551 to 29.048) | 12.007  (7.850 to 17.023) | −1.767  (−1.850 to −1.684) | < 0.001 |
| Costa Rica | 14.378  (9.638 to 20.700) | 9.471  (6.360 to 13.249) | −1.340  (−1.374 to −1.307) | < 0.001 |
| Croatia | 4.415  (2.979 to 6.371) | 3.194  (2.270 to 4.428) | −1.038  (−1.074 to −1.002) | < 0.001 |
| Cuba | 22.633  (14.897 to 32.162) | 15.258  (10.153 to 21.412) | −1.265  (−1.311 to −1.220) | < 0.001 |
| Cyprus | 9.174  (6.200 to 13.180) | 5.451  (3.675 to 7.661) | −1.669  (−1.740 to −1.599) | < 0.001 |
| Czechia | 4.417  (3.009 to 6.270) | 2.981  (2.025 to 4.121) | −1.255  (−1.321 to −1.190) | < 0.001 |
| Democratic People's Republic of Korea | 2.464  (1.673 to 3.509) | 1.578  (1.061 to 2.279) | −1.431  (−1.473 to −1.389) | < 0.001 |
| Democratic Republic of the Congo | 12.352  (8.511 to 16.868) | 12.114  (8.400 to 16.365) | −0.066  (−0.126 to −0.006) | 0.031 |
| Denmark | 6.853  (4.690 to 9.385) | 5.160  (3.562 to 7.236) | −0.914  (−0.998 to −0.830) | < 0.001 |
| Djibouti | 36.486  (24.440 to 51.458) | 26.422  (17.798 to 37.039) | −1.038  (−1.064 to −1.012) | < 0.001 |
| Dominica | 17.457  (11.451 to 24.683) | 11.633  (7.680 to 16.110) | −1.301  (−1.338 to −1.263) | < 0.001 |
| Dominican Republic | 25.149  (16.517 to 35.360) | 15.560  (10.566 to 21.762) | −1.538  (−1.605 to −1.471) | < 0.001 |
| Ecuador | 15.521  (10.269 to 22.089) | 10.001  (6.775 to 13.972) | −1.399  (−1.471 to −1.327) | < 0.001 |
| Egypt | 32.180  (21.570 to 45.871) | 23.460  (15.477 to 32.607) | −1.023  (−1.164 to −0.881) | < 0.001 |
| El Salvador | 24.978  (16.662 to 35.730) | 13.521  (9.268 to 18.244) | −1.967  (−2.004 to −1.930) | < 0.001 |
| Equatorial Guinea | 34.902  (23.116 to 49.888) | 18.322  (11.988 to 25.807) | −2.063  (−2.107 to −2.019) | < 0.001 |
| Eritrea | 41.119  (27.743 to 57.253) | 29.033  (19.661 to 40.839) | −1.110  (−1.168 to −1.053) | < 0.001 |
| Estonia | 4.604  (3.149 to 6.641) | 3.105  (2.096 to 4.254) | −1.262  (−1.335 to −1.189) | < 0.001 |
| Eswatini | 39.277  (25.629 to 56.586) | 27.424  (18.625 to 38.984) | −1.151  (−1.185 to −1.116) | < 0.001 |
| Ethiopia | 42.641  (28.949 to 59.424) | 36.847  (25.311 to 51.769) | −0.472  (−0.543 to −0.401) | < 0.001 |
| Fiji | 17.982  (11.866 to 25.534) | 12.843  (8.358 to 18.093) | −1.085  (−1.294 to −0.875) | < 0.001 |
| Finland | 9.191  (6.227 to 13.007) | 5.931  (3.990 to 8.497) | −1.412  (−1.465 to −1.358) | < 0.001 |
| France | 6.809  (4.596 to 9.675) | 4.702  (3.265 to 6.512) | −1.185  (−1.247 to −1.123) | < 0.001 |
| Gabon | 25.119  (16.722 to 35.778) | 16.489  (11.269 to 23.035) | −1.356  (−1.397 to −1.316) | < 0.001 |
| Gambia | 16.249  (11.381 to 22.115) | 17.763  (11.678 to 25.345) | 0.306  (0.130 to 0.483) | 0.001 |
| Georgia | 12.847  (8.609 to 18.466) | 10.444  (6.993 to 14.720) | −0.673  (−0.700 to −0.646) | < 0.001 |
| Germany | 6.968  (4.794 to 9.897) | 4.838  (3.263 to 6.804) | −1.170  (−1.229 to −1.112) | < 0.001 |
| Ghana | 23.571  (15.836 to 32.931) | 18.245  (12.645 to 24.519) | −0.844  (−0.983 to −0.704) | < 0.001 |
| Greece | 7.774  (5.256 to 11.104) | 5.615  (3.770 to 8.119) | −1.067  (−1.123 to −1.012) | < 0.001 |
| Greenland | 7.118  (4.871 to 9.969) | 6.129  (4.161 to 8.624) | −0.515  (−0.610 to −0.420) | < 0.001 |
| Grenada | 19.747  (12.856 to 28.139) | 11.648  (7.880 to 16.548) | −1.688  (−1.715 to −1.662) | < 0.001 |
| Guam | 10.260  (6.786 to 14.619) | 8.088  (5.353 to 11.436) | −0.823  (−0.989 to −0.657) | < 0.001 |
| Guatemala | 23.240  (15.330 to 32.344) | 13.119  (9.056 to 17.881) | −1.841  (−1.925 to −1.758) | < 0.001 |
| Guinea | 37.180  (24.726 to 52.492) | 34.444  (22.943 to 48.302) | −0.250  (−0.283 to −0.218) | < 0.001 |
| Guinea-Bissau | 32.357  (21.288 to 45.254) | 29.611  (19.891 to 41.670) | −0.268  (−0.307 to −0.228) | < 0.001 |
| Guyana | 22.437  (15.009 to 31.883) | 13.713  (9.208 to 19.331) | −1.576  (−1.611 to −1.540) | < 0.001 |
| Haiti | 30.202  (19.684 to 43.011) | 20.342  (13.493 to 29.101) | −1.265  (−1.313 to −1.217) | < 0.001 |
| Honduras | 20.505  (13.659 to 29.238) | 12.752  (8.622 to 17.648) | −1.527  (−1.566 to −1.488) | < 0.001 |
| Hungary | 8.120  (5.462 to 11.795) | 4.914  (3.372 to 6.880) | −1.594  (−1.647 to −1.542) | < 0.001 |
| Iceland | 7.826  (5.276 to 11.138) | 6.233  (4.091 to 8.997) | −0.732  (−0.782 to −0.681) | < 0.001 |
| India | 20.428  (14.167 to 28.168) | 12.162  (8.539 to 16.621) | −1.652  (−1.906 to −1.397) | < 0.001 |
| Indonesia | 11.917  (7.937 to 16.820) | 7.735  (5.344 to 10.664) | −1.379  (−1.422 to −1.336) | < 0.001 |
| Iran (Islamic Republic of) | 59.015  (40.851 to 81.622) | 36.578  (25.596 to 50.336) | −1.493  (−1.56 to −1.426) | < 0.001 |
| Iraq | 40.808  (26.681 to 57.670) | 22.674  (15.087 to 31.662) | −1.884  (−1.935 to −1.833) | < 0.001 |
| Ireland | 8.238  (5.648 to 11.644) | 5.337  (3.592 to 7.644) | −1.388  (−1.421 to −1.354) | < 0.001 |
| Israel | 8.771  (5.896 to 12.482) | 6.169  (4.203 to 8.742) | −1.136  (−1.190 to −1.082) | < 0.001 |
| Italy | 22.153  (15.188 to 30.989) | 13.909  (9.507 to 19.303) | −1.516  (−1.592 to −1.441) | < 0.001 |
| Ivory Coast | 18.416  (12.118 to 25.584) | 29.133  (19.555 to 40.67) | 1.508  (1.308 to 1.709) | < 0.001 |
| Jamaica | 16.893  (11.176 to 24.501) | 11.476  (7.805 to 16.295) | −1.240  (−1.297 to −1.183) | < 0.001 |
| Japan | 7.382  (5.141 to 10.177) | 5.893  (4.081 to 8.076) | −0.721  (−0.744 to −0.698) | < 0.001 |
| Jordan | 36.340  (24.160 to 52.114) | 18.953  (12.835 to 26.360) | −2.075  (−2.134 to −2.016) | < 0.001 |
| Kazakhstan | 14.348  (9.610 to 20.731) | 9.918  (6.828 to 13.889) | −1.189  (−1.250 to −1.128) | < 0.001 |
| Kenya | 30.155  (20.826 to 41.708) | 24.470  (16.910 to 33.616) | −0.681  (−0.788 to −0.574) | < 0.001 |
| Kiribati | 15.406  (10.253 to 21.903) | 12.660  (8.242 to 17.912) | −0.663  (−0.767 to −0.558) | < 0.001 |
| Kuwait | 27.052  (17.912 to 38.025) | 16.794  (11.503 to 23.429) | −1.527  (−1.571 to −1.482) | < 0.001 |
| Kyrgyzstan | 17.824  (11.876 to 25.861) | 13.795  (9.326 to 19.396) | −0.816  (−0.881 to −0.750) | < 0.001 |
| Lao People's Democratic Republic | 8.343  (5.672 to 11.705) | 5.440  (3.642 to 7.672) | −1.371  (−1.416 to −1.326) | < 0.001 |
| Latvia | 6.900  (4.670 to 9.877) | 4.925  (3.352 to 6.905) | −1.082  (−1.146 to −1.018) | < 0.001 |
| Lebanon | 30.234  (20.708 to 42.295) | 17.937  (11.905 to 24.84) | −1.678  (−1.735 to −1.621) | < 0.001 |
| Lesotho | 41.460  (27.569 to 59.143) | 31.309  (20.645 to 43.986) | −0.902  (−0.933 to −0.872) | < 0.001 |
| Liberia | 39.458  (26.223 to 55.480) | 27.785  (18.459 to 38.557) | −1.128  (−1.191 to −1.066) | < 0.001 |
| Libya | 55.072  (36.562 to 77.145) | 32.375  (21.825 to 45.135) | −1.441  (−1.816 to −1.065) | < 0.001 |
| Lithuania | 7.004  (4.723 to 9.844) | 4.824  (3.316 to 6.903) | −1.205  (−1.28 to −1.129) | < 0.001 |
| Luxembourg | 7.614  (5.123 to 10.756) | 5.045  (3.405 to 7.167) | −1.325  (−1.365 to −1.286) | < 0.001 |
| Madagascar | 30.160  (20.945 to 41.719) | 22.167  (15.333 to 30.101) | −0.988  (−1.018 to −0.958) | < 0.001 |
| Malawi | 47.574  (33.326 to 64.074) | 29.180  (19.564 to 40.524) | −1.595  (−1.653 to −1.537) | < 0.001 |
| Malaysia | 7.211  (4.822 to 10.152) | 5.567  (3.765 to 7.951) | −0.841  (−0.943 to −0.740) | < 0.001 |
| Maldives | 15.695  (10.214 to 22.394) | 6.929  (4.747 to 9.615) | −2.619  (−2.718 to −2.521) | < 0.001 |
| Mali | 40.440  (28.100 to 54.825) | 34.897  (24.116 to 48.783) | −0.465  (−0.519 to −0.412) | < 0.001 |
| Malta | 8.851  (5.900 to 12.733) | 5.778  (3.878 to 8.207) | −1.372  (−1.407 to −1.337) | < 0.001 |
| Marshall Islands | 15.587  (10.269 to 22.214) | 10.814  (7.174 to 15.366) | −1.167  (−1.243 to −1.091) | < 0.001 |
| Mauritania | 34.112  (23.009 to 47.328) | 26.126  (17.621 to 36.944) | −0.857  (−0.884 to −0.830) | < 0.001 |
| Mauritius | 9.566  (6.446 to 13.478) | 5.888  (4.033 to 8.127) | −1.550  (−1.588 to −1.512) | < 0.001 |
| Mexico | 17.417  (12.110 to 23.534) | 11.194  (7.850 to 14.829) | −1.430  (−1.460 to −1.400) | < 0.001 |
| Micronesia (Federated States of) | 15.975  (10.742 to 22.809) | 11.252  (7.230 to 16.108) | −1.156  (−1.305 to −1.007) | < 0.001 |
| Monaco | 6.521  (4.362 to 9.380) | 5.256  (3.507 to 7.480) | −0.702  (−0.738 to −0.667) | < 0.001 |
| Mongolia | 26.067  (17.597 to 37.406) | 15.697  (10.656 to 22.367) | −1.634  (−1.706 to −1.561) | < 0.001 |
| Montenegro | 4.347  (2.885 to 6.194) | 3.402  (2.317 to 4.833) | −0.802  (−0.849 to −0.756) | < 0.001 |
| Morocco | 27.080  (19.198 to 36.749) | 17.460  (12.122 to 24.047) | −1.402  (−1.455 to −1.350) | < 0.001 |
| Mozambique | 33.956  (22.535 to 47.491) | 24.804  (17.092 to 33.845) | −1.026  (−1.081 to −0.971) | < 0.001 |
| Myanmar | 16.318  (10.449 to 23.167) | 10.360  (6.589 to 14.628) | −1.466  (−1.705 to −1.227) | < 0.001 |
| Namibia | 38.415  (25.339 to 55.152) | 25.834  (17.210 to 36.691) | −1.278  (−1.307 to −1.248) | < 0.001 |
| Nauru | 15.195  (10.038 to 21.557) | 10.829  (7.096 to 15.384) | −1.124  (−1.223 to −1.025) | < 0.001 |
| Nepal | 13.827  (9.827 to 18.726) | 6.927  (4.788 to 9.578) | −2.204  (−2.353 to −2.056) | < 0.001 |
| Netherlands | 6.322  (4.348 to 9.038) | 4.778  (3.191 to 6.768) | −0.906  (−0.937 to −0.874) | < 0.001 |
| New Zealand | 7.225  (5.062 to 10.089) | 5.347  (3.720 to 7.387) | −0.964  (−1 to −0.928) | < 0.001 |
| Nicaragua | 20.709  (13.960 to 29.443) | 12.756  (8.529 to 17.834) | −1.557  (−1.581 to −1.533) | < 0.001 |
| Niger | 41.529  (27.239 to 58.468) | 41.825  (26.976 to 58.469) | 0.020  (−0.010 to 0.049) | 0.188 |
| Nigeria | 49.080  (34.630 to 67.603) | 36.434  (25.359 to 50.239) | −0.963  (−1.047 to −0.879) | < 0.001 |
| Niue | 12.189  (8.109 to 17.628) | 8.295  (5.517 to 11.655) | −1.262  (−1.326 to −1.198) | < 0.001 |
| North Macedonia | 5.730  (3.879 to 8.253) | 3.752  (2.52 to 5.371) | −1.355  (−1.402 to −1.308) | < 0.001 |
| Northern Mariana Islands | 10.410  (6.865 to 14.798) | 8.078  (5.301 to 11.564) | −0.847  (−0.902 to −0.792) | < 0.001 |
| Norway | 5.826  (4.063 to 8.028) | 4.745  (3.330 to 6.556) | −0.659  (−0.702 to −0.615) | < 0.001 |
| Oman | 35.648  (23.672 to 50.662) | 21.905  (14.887 to 30.685) | −1.548  (−1.616 to −1.481) | < 0.001 |
| Pakistan | 12.864  (8.954 to 17.371) | 13.421  (9.398 to 18.643) | 0.122  (0.073 to 0.172) | < 0.001 |
| Palau | 11.899  (7.870 to 16.762) | 8.649  (5.747 to 11.966) | −1.048  (−1.131 to −0.965) | < 0.001 |
| Palestine | 29.443  (19.268 to 41.611) | 16.459  (10.963 to 22.975) | −1.885  (−1.982 to −1.787) | < 0.001 |
| Panama | 16.837  (11.059 to 24.129) | 10.798  (7.251 to 15.065) | −1.431  (−1.479 to −1.382) | < 0.001 |
| Papua New Guinea | 12.800  (8.398 to 17.975) | 10.377  (6.917 to 14.701) | −0.673  (−0.761 to −0.585) | < 0.001 |
| Paraguay | 20.003  (13.852 to 27.311) | 13.869  (9.599 to 18.983) | −1.183  (−1.223 to −1.142) | < 0.001 |
| Peru | 25.886  (17.258 to 36.916) | 15.531  (10.501 to 21.769) | −1.634  (−1.681 to −1.586) | < 0.001 |
| Philippines | 11.491  (7.925 to 15.885) | 9.081  (6.265 to 12.552) | −0.755  (−0.783 to −0.726) | < 0.001 |
| Poland | 4.391  (3.075 to 5.969) | 3.347  (2.315 to 4.512) | −0.868  (−0.907 to −0.829) | < 0.001 |
| Portugal | 10.286  (6.878 to 14.572) | 6.169  (4.202 to 8.728) | −1.645  (−1.716 to −1.575) | < 0.001 |
| Puerto Rico | 13.642  (9.063 to 19.444) | 8.318  (5.693 to 11.596) | −1.580  (−1.605 to −1.554) | < 0.001 |
| Qatar | 48.590  (32.803 to 68.235) | 24.279  (16.390 to 34.063) | −2.310  (−2.603 to −2.017) | < 0.001 |
| Republic of Korea | 12.507  (8.431 to 17.374) | 6.365  (4.354 to 8.908) | −2.174  (−2.230 to −2.118) | < 0.001 |
| Republic of Moldova | 8.338  (5.604 to 11.777) | 5.748  (4.030 to 7.728) | −1.188  (−1.256 to −1.119) | < 0.001 |
| Romania | 5.774  (3.895 to 8.272) | 3.878  (2.630 to 5.629) | −1.272  (−1.354 to −1.190) | < 0.001 |
| Russian Federation | 11.179  (7.756 to 15.414) | 7.475  (5.176 to 10.128) | −1.291  (−1.315 to −1.266) | < 0.001 |
| Rwanda | 28.136  (19.292 to 38.713) | 18.087  (12.644 to 24.699) | −1.413  (−1.453 to −1.372) | < 0.001 |
| Saint Kitts and Nevis | 18.411  (11.962 to 26.423) | 10.992  (7.326 to 15.572) | −1.648  (−1.687 to −1.609) | < 0.001 |
| Saint Lucia | 18.431  (12.217 to 26.241) | 11.273  (7.484 to 15.985) | −1.566  (−1.609 to −1.523) | < 0.001 |
| Saint Vincent and the Grenadines | 19.194  (12.727 to 27.305) | 12.665  (8.419 to 17.807) | −1.328  (−1.374 to −1.282) | < 0.001 |
| Samoa | 13.481  (8.963 to 19.931) | 10.025  (6.607 to 14.257) | −0.979  (−1.046 to −0.913) | < 0.001 |
| San Marino | 6.606  (4.429 to 9.414) | 5.357  (3.651 to 7.632) | −0.680  (−0.716 to −0.643) | < 0.001 |
| Sao Tome and Principe | 37.097  (24.367 to 52.337) | 25.886  (17.328 to 35.817) | −1.157  (−1.189 to −1.125) | < 0.001 |
| Saudi Arabia | 46.209  (32.586 to 62.932) | 28.576  (19.071 to 40.527) | −1.552  (−1.623 to −1.482) | < 0.001 |
| Senegal | 38.977  (26.169 to 54.003) | 28.888  (19.482 to 39.409) | −0.930  (−1.038 to −0.821) | < 0.001 |
| Serbia | 5.300  (3.559 to 7.706) | 3.535  (2.389 to 4.906) | −1.282  (−1.322 to −1.241) | < 0.001 |
| Seychelles | 10.014  (6.668 to 14.367) | 6.203  (4.170 to 8.533) | −1.531  (−1.591 to −1.470) | < 0.001 |
| Sierra Leone | 38.897  (24.899 to 56.206) | 30.354  (20.452 to 42.980) | −0.815  (−1.008 to −0.622) | < 0.001 |
| Singapore | 11.223  (7.574 to 15.789) | 7.847  (5.269 to 10.910) | −1.145  (−1.205 to −1.085) | < 0.001 |
| Slovakia | 4.896  (3.316 to 6.967) | 3.477  (2.387 to 4.966) | −1.103  (−1.146 to −1.061) | < 0.001 |
| Slovenia | 4.120  (2.783 to 6.004) | 2.943  (1.960 to 4.148) | −1.074  (−1.144 to −1.003) | < 0.001 |
| Solomon Islands | 18.684  (12.192 to 26.597) | 13.855  (9.192 to 19.389) | −0.984  (−1.073 to −0.895) | < 0.001 |
| Somalia | 30.867  (20.704 to 43.630) | 29.350  (19.710 to 42.076) | −0.164  (−0.199 to −0.128) | < 0.001 |
| South Africa | 29.776  (20.565 to 41.775) | 23.626  (16.327 to 32.697) | −0.727  (−0.944 to −0.509) | < 0.001 |
| South Sudan | 25.826  (16.764 to 36.685) | 18.774  (12.009 to 26.917) | −0.972  (−1.057 to −0.887) | < 0.001 |
| Spain | 19.449  (13.233 to 27.871) | 12.691  (8.678 to 17.659) | −1.372  (−1.424 to −1.32) | < 0.001 |
| Sri Lanka | 8.652  (5.960 to 12.455) | 5.121  (3.573 to 7.059) | −1.670  (−1.741 to −1.600) | < 0.001 |
| Sudan | 53.052  (35.548 to 74.542) | 27.981  (18.523 to 38.501) | −1.949  (−2.032 to −1.866) | < 0.001 |
| Suriname | 23.728  (15.738 to 33.198) | 16.048  (10.932 to 21.913) | −1.260  (−1.297 to −1.222) | < 0.001 |
| Sweden | 2.970  (2.053 to 4.239) | 2.696  (1.838 to 3.842) | −0.253  (−0.312 to −0.195) | < 0.001 |
| Switzerland | 6.137  (4.163 to 8.670) | 4.773  (3.236 to 6.822) | −0.811  (−0.846 to −0.777) | < 0.001 |
| Syrian Arab Republic | 45.084  (30.129 to 63.241) | 24.190  (16.452 to 33.802) | −1.985  (−2.059 to −1.912) | < 0.001 |
| Taiwan (Province of China) | 1.024  (0.637 to 1.559) | 0.589  (0.356 to 0.912) | −1.788  (−1.904 to −1.672) | < 0.001 |
| Tajikistan | 18.851  (12.505 to 27.265) | 15.140  (10.174 to 21.291) | −0.699  (−0.753 to −0.646) | < 0.001 |
| Thailand | 8.389  (5.795 to 11.381) | 4.339  (2.990 to 6.160) | −2.124  (−2.228 to −2.020) | < 0.001 |
| Timor-Leste | 29.651  (19.577 to 42.078) | 14.981  (10.078 to 21.240) | −2.119  (−2.188 to −2.051) | < 0.001 |
| Togo | 27.505  (18.443 to 37.980) | 27.644  (18.393 to 38.849) | 0.015  (−0.024 to 0.054) | 0.451 |
| Tokelau | 14.983  (9.757 to 21.691) | 9.747  (6.438 to 13.971) | −1.403  (−1.465 to −1.342) | < 0.001 |
| Tonga | 13.396  (9.214 to 18.872) | 8.654  (5.657 to 12.124) | −1.419  (−1.618 to −1.220) | < 0.001 |
| Trinidad and Tobago | 16.305  (10.753 to 23.244) | 9.713  (6.686 to 13.579) | −1.660  (−1.704 to −1.616) | < 0.001 |
| Tunisia | 44.702  (30.289 to 63.756) | 21.19  (14.138 to 29.751) | −2.386  (−2.407 to −2.365) | < 0.001 |
| Turkey | 25.664  (17.188 to 36.335) | 13.664  (9.191 to 19.585) | −2.026  (−2.067 to −1.985) | < 0.001 |
| Turkmenistan | 11.446  (7.774 to 15.818) | 8.443  (5.729 to 12.145) | −0.980  (−1.027 to −0.933) | < 0.001 |
| Tuvalu | 16.173  (10.555 to 22.636) | 11.038  (7.329 to 15.626) | −1.243  (−1.351 to −1.134) | < 0.001 |
| Uganda | 41.460  (28.483 to 56.709) | 19.909  (13.951 to 27.297) | −2.211  (−2.494 to −1.927) | < 0.001 |
| Ukraine | 7.242  (4.998 to 10.028) | 5.893  (3.997 to 8.048) | −0.655  (−0.710 to −0.600) | < 0.001 |
| United Arab Emirates | 34.835  (23.344 to 49.251) | 21.033  (14.505 to 29.91) | −1.613  (−1.655 to −1.572) | < 0.001 |
| United Kingdom | 7.252  (5.016 to 10.078) | 5.517  (3.861 to 7.622) | −0.883  (−0.903 to −0.863) | < 0.001 |
| United Republic of Tanzania | 45.007  (30.502 to 62.592) | 31.064  (21.299 to 43.218) | −1.195  (−1.393 to −0.997) | < 0.001 |
| United States of America | 5.092  (3.533 to 6.952) | 4.593  (3.202 to 6.290) | −0.327  (−0.400 to −0.254) | < 0.001 |
| United States Virgin Islands | 14.599  (9.659 to 20.683) | 9.776  (6.506 to 13.658) | −1.286  (−1.316 to −1.256) | < 0.001 |
| Uruguay | 7.218  (4.931 to 10.056) | 5.571  (3.767 to 7.698) | −0.835  (−0.863 to −0.806) | < 0.001 |
| Uzbekistan | 17.079  (11.395 to 24.191) | 12.371  (8.322 to 17.748) | −1.041  (−1.094 to −0.988) | < 0.001 |
| Vanuatu | 11.842  (8.250 to 16.703) | 8.317  (5.509 to 11.778) | −1.162  (−1.279 to −1.045) | < 0.001 |
| Venezuela (Bolivarian Republic of) | 15.495  (10.585 to 21.029) | 11.320  (7.811 to 15.685) | −0.996  (−1.083 to −0.909) | < 0.001 |
| Viet Nam | 11.527  (7.933 to 15.959) | 6.558  (4.621 to 9.028) | −1.815  (−1.875 to −1.756) | < 0.001 |
| Yemen | 26.392  (17.675 to 37.425) | 21.839  (14.783 to 30.884) | −0.618  (−0.716 to −0.520) | < 0.001 |
| Zambia | 35.612  (24.075 to 49.602) | 24.446  (16.462 to 34.123) | −1.147  (−1.254 to −1.041) | < 0.001 |
| Zimbabwe | 23.913  (16.473 to 33.792) | 20.168  (13.713 to 27.634) | −0.552  (−0.584 to −0.521) | < 0.001 |

ASYR = age-standardized years lived with disability rate per 100,000 populations; UI = uncertainty interval; AAPC = average annual percent change; CI = confidence interval

**Supplementary Table S6. Multivariable linear regression analysis of the associations between SDI and the ASYR of AREDs.**

| Variables | Estimate | SE | 95% CI | t value | *P* value |
| --- | --- | --- | --- | --- | --- |
| ASYR of AMD |  |  |  |  |  |
| SDI | −0.350 | 0.037 | −0.423 to −0.277 | −9.571 | < 0.001 |
| Smoking | 0.817 | 0.037 | 0.744 to 0.890 | 22.346 | < 0.001 |
| ASYR of cataract |  |  |  |  |  |
| SDI | −0.134 | 0.037 | −0.207 to −0.061 | −3.600 | < 0.001 |
| Smoking | 0.134 | 0.029 | 0.077 to 0.191 | 4.627 | < 0.001 |
| Household air pollution from solid fuels | 0.119 | 0.037 | 0.046 to 0.192 | 3.168 | 0.002 |
| High body-mass index | 0.028 | 0.037 | −0.045 to 0.101 | 0.773 | 0.441 |
| High fasting plasma glucose | 0.687 | 0.046 | 0.597 to 0.777 | 14.834 | < 0.001 |
| ASYR of glaucoma |  |  |  |  |  |
| SDI | −0.213 | 0.029 | −0.270 to −0.156 | −7.112 | < 0.001 |
| High fasting plasma glucose | 0.796 | 0.029 | 0.739 to 0.853 | 26.606 | < 0.001 |

SDI = socio-demographic index; ASYR = age-standardized years lived with disability rate; AREDs = age-related eye diseases; CI = confidence interval; SE = standard error; AMD = age-related macular degeneration; AREDs = age-related eye diseases

**Supplementary Table S7. Global slope index of inequality (SII) in the Burden of Age-Related Eye Diseases (1990–2021).**

| Year | AMD | Cataract | Glaucoma |
| --- | --- | --- | --- |
| 1990 | −9.250  (−11.590 to −6.909) | −258.131  (−291.377 to −224.885) | −21.090  (−25.597 to −16.583) |
| 1991 | −8.525  (−10.878 to −6.173) | −254.467  (−287.863 to −221.071) | −19.941  (−24.618 to −15.265) |
| 1992 | −8.501  (−10.835 to −6.168) | −251.890  (−285.321 to −218.459) | −20.319  (−25.025 to −15.614) |
| 1993 | −8.864  (−11.160 to −6.568) | −234.151  (−269.643 to −198.658) | −20.878  (−25.574 to −16.181) |
| 1994 | −8.871  (−11.155 to −6.587) | −231.712  (−267.175 to −196.250) | −21.727  (−26.361 to −17.093) |
| 1995 | −8.607  (−10.896 to −6.319) | −229.379  (−264.693 to −194.065) | −22.017  (−26.607 to −17.427) |
| 1996 | −8.032  (−10.316 to −5.748) | −243.265  (−276.220 to −210.311) | −22.143  (−26.621 to −17.665) |
| 1997 | −7.747  (−9.972 to −5.521) | −241.909  (−274.496 to −209.321) | −21.633  (−25.967 to −17.3) |
| 1998 | −7.503  (−9.662 to −5.344) | −240.727  (−272.965 to −208.489) | −21.076  (−25.245 to −16.907) |
| 1999 | −6.862  (−8.995 to −4.729) | −239.996  (−271.862 to −208.129) | −21.040  (−25.032 to −17.049) |
| 2000 | −6.695  (−8.801 to −4.589) | −240.798  (−272.093 to −209.503) | −20.730  (−24.654 to −16.805) |
| 2001 | −6.627  (−8.721 to −4.534) | −238.784  (−269.831 to −207.738) | −20.599  (−24.511 to −16.687) |
| 2002 | −6.66  (−8.739 to −4.582) | −237.579  (−268.140 to −207.017) | −20.693  (−24.591 to −16.795) |
| 2003 | −6.601  (−8.671 to −4.53) | −235.058  (−265.294 to −204.823) | −20.744  (−24.645 to −16.842) |
| 2004 | −6.535  (−8.602 to −4.468) | −232.837  (−262.728 to −202.947) | −20.742  (−24.652 to −16.833) |
| 2005 | −6.303  (−8.376 to −4.229) | −230.345  (−260.028 to −200.662) | −20.972  (−24.849 to −17.096) |
| 2006 | −6.149  (−8.223 to −4.076) | −227.650  (−257.178 to −198.122) | −20.865  (−24.713 to −17.016) |
| 2007 | −6.019  (−8.088 to −3.949) | −224.663  (−254.004 to −195.323) | −20.816  (−24.606 to −17.025) |
| 2008 | −5.854  (−7.923 to −3.784) | −221.357  (−250.53 to −192.184) | −20.564  (−24.306 to −16.821) |
| 2009 | −5.612  (−7.693 to −3.532) | −219.221  (−248.083 to −190.359) | −20.286  (−23.99 to −16.583) |
| 2010 | −5.479  (−7.565 to −3.392) | −216.920  (−245.55 to −188.291) | −20.317  (−23.966 to −16.669) |
| 2011 | −5.884  (−7.946 to −3.821) | −214.137  (−242.582 to −185.692) | −20.967  (−24.495 to −17.44) |
| 2012 | −5.808  (−7.865 to −3.752) | −211.151  (−239.372 to −182.93) | −20.639  (−24.145 to −17.134) |
| 2013 | −5.781  (−7.828 to −3.734) | −208.365  (−236.322 to −180.409) | −20.364  (−23.844 to −16.883) |
| 2014 | −5.754  (−7.792 to −3.716) | −203.945  (−231.862 to −176.028) | −20.109  (−23.566 to −16.652) |
| 2015 | −5.809  (−7.837 to −3.781) | −200.715  (−228.447 to −172.983) | −20.001  (−23.437 to −16.565) |
| 2016 | −5.981  (−7.979 to −3.983) | −194.797  (−222.221 to −167.372) | −19.967  (−23.387 to −16.548) |
| 2017 | −6.17  (−8.122 to −4.217) | −188.736  (−215.331 to −162.141) | −19.905  (−23.323 to −16.486) |
| 2018 | −6.335  (−8.248 to −4.422) | −181.609  (−207.612 to −155.605) | −19.994  (−23.407 to −16.581) |
| 2019 | −6.426  (−8.317 to −4.534) | −177.652  (−203.281 to −152.023) | −20.053  (−23.463 to −16.642) |
| 2020 | −6.271  (−8.200 to −4.342) | −176.204  (−202.092 to −150.317) | −20.421  (−23.857 to −16.985) |
| 2021 | −6.033  (−7.976 to −4.090) | −173.762  (−199.899 to −147.624) | −20.064  (−23.475 to −16.653) |

AMD = age-related macular degeneration.

**Supplementary Table S8. Global concentration index (CI) in the Burden of Age-Related Eye Diseases (1990–2021).**

| Year | AMD | Cataract | Glaucoma |
| --- | --- | --- | --- |
| 1990 | −0.167  (−0.247 to −0.086) | −0.335  (−0.432 to −0.237) | −0.208  (−0.302 to −0.114) |
| 1991 | −0.151  (−0.238 to −0.063) | −0.334  (−0.430 to −0.238) | −0.200  (−0.298 to −0.101) |
| 1992 | −0.148  (−0.233 to −0.063) | −0.334  (−0.428 to −0.239) | −0.206  (−0.303 to −0.110) |
| 1993 | −0.153  (−0.237 to −0.069) | −0.312  (−0.429 to −0.195) | −0.215  (−0.313 to −0.116) |
| 1994 | −0.153  (−0.240 to −0.065) | −0.310  (−0.429 to −0.191) | −0.226  (−0.320 to −0.132) |
| 1995 | −0.148  (−0.238 to −0.059) | −0.308  (−0.423 to −0.193) | −0.230  (−0.324 to −0.136) |
| 1996 | −0.140  (−0.229 to −0.051) | −0.326  (−0.420 to −0.232) | −0.232  (−0.323 to −0.141) |
| 1997 | −0.138  (−0.225 to −0.051) | −0.322  (−0.415 to −0.228) | −0.228  (−0.317 to −0.139) |
| 1998 | −0.137  (−0.226 to −0.049) | −0.317  (−0.408 to −0.226) | −0.224  (−0.313 to −0.135) |
| 1999 | −0.128  (−0.218 to −0.039) | −0.313  (−0.408 to −0.219) | −0.225  (−0.311 to −0.139) |
| 2000 | −0.127  (−0.218 to −0.035) | −0.314  (−0.408 to −0.219) | −0.223  (−0.311 to −0.135) |
| 2001 | −0.126  (−0.214 to −0.037) | −0.312  (−0.402 to −0.222) | −0.223  (−0.311 to −0.136) |
| 2002 | −0.127  (−0.223 to −0.030) | −0.312  (−0.405 to −0.219) | −0.227  (−0.320 to −0.133) |
| 2003 | −0.126  (−0.222 to −0.030) | −0.311  (−0.402 to −0.220) | −0.230  (−0.320 to −0.139) |
| 2004 | −0.125  (−0.219 to −0.031) | −0.310  (−0.402 to −0.219) | −0.232  (−0.323 to −0.141) |
| 2005 | −0.121  (−0.218 to −0.024) | −0.309  (−0.397 to −0.221) | −0.237  (−0.327 to −0.147) |
| 2006 | −0.119  (−0.216 to −0.022) | −0.308  (−0.399 to −0.217) | −0.238  (−0.332 to −0.145) |
| 2007 | −0.118  (−0.216 to −0.021) | −0.307  (−0.399 to −0.215) | −0.241  (−0.333 to −0.149) |
| 2008 | −0.117  (−0.212 to −0.021) | −0.306  (−0.400 to −0.211) | −0.242  (−0.336 to −0.148) |
| 2009 | −0.114  (−0.213 to −0.014) | −0.306  (−0.400 to −0.211) | −0.242  (−0.335 to −0.150) |
| 2010 | −0.112  (−0.214 to −0.010) | −0.305  (−0.396 to −0.214) | −0.245  (−0.338 to −0.152) |
| 2011 | −0.121  (−0.217 to −0.025) | −0.303  (−0.397 to −0.209) | −0.255  (−0.352 to −0.158) |
| 2012 | −0.121  (−0.215 to −0.026) | −0.301  (−0.393 to −0.208) | −0.254  (−0.353 to −0.154) |
| 2013 | −0.121  (−0.216 to −0.026) | −0.298  (−0.390 to −0.207) | −0.253  (−0.351 to −0.155) |
| 2014 | −0.121  (−0.214 to −0.029) | −0.294  (−0.389 to −0.199) | −0.252  (−0.349 to −0.154) |
| 2015 | −0.123  (−0.217 to −0.030) | −0.291  (−0.386 to −0.196) | −0.252  (−0.350 to −0.154) |
| 2016 | −0.128  (−0.216 to −0.039) | −0.286  (−0.382 to −0.191) | −0.254  (−0.346 to −0.162) |
| 2017 | −0.133  (−0.218 to −0.047) | −0.284  (−0.377 to −0.190) | −0.255  (−0.351 to −0.159) |
| 2018 | −0.137  (−0.219 to −0.054) | −0.279  (−0.374 to −0.185) | −0.258  (−0.354 to −0.161) |
| 2019 | −0.139  (−0.218 to −0.059) | −0.277  (−0.370 to −0.183) | −0.259  (−0.350 to −0.169) |
| 2020 | −0.134  (−0.218 to −0.049) | −0.272  (−0.364 to −0.180) | −0.264  (−0.363 to −0.166) |
| 2021 | −0.129  (−0.217 to −0.041) | −0.272  (−0.375 to −0.169) | −0.263  (−0.369 to −0.157) |

AMD = age-related macular degeneration.

**Supplementary Table S9. Regional slope index of inequality (SII) in the Burden of Age-Related Eye Diseases in 1990 and 2021.**

| GBD region | SII of AMD | | SII of cataract | | SII of glaucoma | |
| --- | --- | --- | --- | --- | --- | --- |
|  | 1990 | 2021 | 1990 | 2021 | 1990 | 2021 |
| Andean Latin America | −8.392  (−37.790 to 21.006) | 13.191  (−2.823 to 29.204) | −98.477  (−298.537 to 101.583) | 36.332  (−74.126 to 146.789) | −14.453  (−33.801 to 4.895) | 3.912  (−13.241 to 21.065) |
| Caribbean | −1.023  (−1.626 to −0.420) | −0.688  (−1.137 to −0.239) | −25.907  (−46.305 to −5.509) | −19.332  (−32.013 to −6.651) | −15.946  (−20.467 to −11.424) | −12.166  (−14.543 to −9.789) |
| Central Asia | −0.369  (−2.445 to 1.706) | −1.291  (−3.372 to 0.79) | −6.246  (−48.097 to 35.606) | −2.379  (−33.448 to 28.691) | −6.605  (−11.482 to −1.727) | −6.003  (−8.279 to −3.727) |
| Central Europe | −0.388  (−1.486 to 0.710) | −0.518  (−1.249 to 0.213) | −0.682  (−10.466 to 9.103) | −0.698  (−7.896 to 6.499) | −0.764  (−3.136 to 1.608) | −0.875  (−1.883 to 0.132) |
| Central Latin America | −1.600  (−3.275 to 0.076) | 0.062  (−1.049 to 1.173) | 1.536  (−29.492 to 32.564) | 26.248  (9.095 to 43.401) | −6.309  (−9.894 to −2.724) | −1.867  (−3.409 to −0.324) |
| Central Sub-Saharan Africa | −1.585  (−4.555 to 1.384) | 1.984  (1.170 to 2.798) | −82.154  (−207.259 to 42.951) | 51.655  (9.410 to 93.900) | −21.029  (−48.046 to 5.988) | 12.676  (3.371 to 21.981) |
| East Asia | −11.363  (−13.746 to −8.979) | −0.289  (−22.704 to 22.126) | −100.691  (−125.517 to −75.865) | −3.876  (−168.533 to 160.781) | −17.505  (−19.789 to −15.22) | −0.585  (−15.574 to 14.405) |
| Eastern Europe | −0.238  (−0.887 to 0.411) | −0.58  (−1.107 to −0.053) | −17.462  (−32.432 to −2.492) | −18.389  (−31.197 to −5.582) | 6.361  (2.367 to 10.354) | 2.333  (−0.378 to 5.043) |
| Eastern Sub-Saharan Africa | 16.067  (3.090 to 29.044) | 2.011  (−4.354 to 8.375) | −103.444  (−235.177 to 28.288) | −73.395  (−195.508 to 48.718) | −10.088  (−22.693 to 2.516) | −5.538  (−17.469 to 6.394) |
| High-income Asia Pacific | −2.586  (−3.407 to −1.765) | 0.228  (−0.529 to 0.985) | −15.494  (−17.303 to −13.684) | 1.895  (−5.407 to 9.197) | −9.939  (−12.136 to −7.742) | 0.301  (−1.948 to 2.550) |
| High-income North America | −0.672  (−0.744 to −0.599) | −0.339  (−0.407 to −0.271) | −1.122  (−1.413 to −0.831) | −0.812  (−0.993 to −0.631) | −0.399  (−0.742 to −0.055) | −0.430  (−0.645 to −0.215) |
| North Africa and Middle East | 4.497  (−6.418 to 15.411) | 1.043  (−7.176 to 9.262) | 12.473  (−83.885 to 108.831) | −31.195  (−83.985 to 21.595) | −0.525  (−19.474 to 18.424) | −3.140  (−14.054 to 7.774) |
| Oceania | 2.739  (1.232 to 4.246) | 2.497  (1.94 to 3.055) | −114.208  (−175.494 to −52.922) | −146.914  (−167.425 to −126.402) | 1.744  (−2.700 to 6.189) | 2.248  (−0.177 to 4.674) |
| South Asia | 1.913  (−5.919 to 9.745) | −4.559  (−14.249 to 5.131) | 19.480  (−146.475 to 185.435) | −76.174  (−290.5 to 138.152) | 12.657  (8.466 to 16.847) | 3.393  (−3.347 to 10.133) |
| Southeast Asia | 2.46  (−3.544 to 8.464) | −0.544  (−3.761 to 2.673) | −86.221  (−203.867 to 31.425) | −5.293  (−81.747 to 71.162) | −5.444  (−8.953 to −1.935) | −3.548  (−6.332 to −0.763) |
| Southern Latin America | 1.494  (−0.008 to 2.996) | −1.128  (−1.881 to −0.375) | −16.732  (−98.845 to 65.381) | 29.337  (20.427 to 38.246) | 1.174  (−1.012 to 3.361) | −0.256  (−1.239 to 0.727) |
| Southern Sub-Saharan Africa | 0.732  (0.291 to 1.173) | 1.726  (1.228 to 2.223) | −22.964  (−43.058 to −2.870) | −23.980  (−43.836 to −4.123) | −0.479  (−27.491 to 26.532) | 1.662  (−12.001 to 15.326) |
| Western Europe | −13.131  (−18.443 to −7.819) | −8.266  (−11.46 to −5.072) | −30.712  (−42.175 to −19.249) | −25.740  (−35.258 to −16.223) | −15.825  (−22.066 to −9.583) | −9.200  (−12.896 to −5.505) |
| Western Sub-Saharan Africa | 15.942  (6.248 to 25.636) | 11.657  (5.594 to 17.719) | 123.359  (10.865 to 235.853) | 95.447  (11.324 to 179.570) | 10.943  (−6.767 to 28.654) | −3.441  (−13.463 to 6.581) |

GBD = global burden of disease; AMD = age-related macular degeneration

**Supplementary Table S10.** Regional concentration index (CI) in the Burden of Age-Related Eye Diseases in 1990 and 2021.

| GBD region | Concentration index of AMD | | Concentration index of cataract | | Concentration index of glaucoma | |
| --- | --- | --- | --- | --- | --- | --- |
|  | 1990 | 2021 | 1990 | 2021 | 1990 | 2021 |
| Andean Latin America | −0.121  (−0.377 to 0.135) | 0.227  (−0.106 to 0.561) | −0.091  (−0.218 to 0.037) | 0.053  (−0.076 to 0.182) | −0.083  (−0.177 to 0.012) | 0.038  (−0.087 to 0.163) |
| Caribbean | −0.070  (−0.132 to −0.009) | −0.059  (−0.118 to 0.001) | −0.068  (−0.169 to 0.033) | −0.069  (−0.149 to 0.010) | −0.111  (−0.190 to −0.033) | −0.123  (−0.194 to −0.052) |
| Central Asia | −0.011  (−0.054 to 0.031) | −0.042  (−0.107 to 0.022) | −0.013  (−0.071 to 0.046) | −0.006  (−0.06 to 0.048) | −0.066  (−0.108 to −0.024) | −0.079  (−0.116 to −0.042) |
| Central Europe | −0.013  (−0.05 to 0.024) | −0.020  (−0.052 to 0.011) | −0.005  (−0.066 to 0.057) | −0.006  (−0.064 to 0.052) | −0.024  (−0.096 to 0.049) | −0.039  (−0.086 to 0.008) |
| Central Latin America | −0.045  (−0.114 to 0.023) | 0.002  (−0.050 to 0.055) | 0.002  (−0.051 to 0.056) | 0.059  (0.003 to 0.116) | −0.050  (−0.092 to −0.008) | −0.023  (−0.048 to 0.001) |
| Central Sub-Saharan Africa | −0.087  (−0.272 to 0.098) | 0.114  (0.025 to 0.202) | −0.268  (−0.592 to 0.057) | 0.216  (0.030 to 0.402) | −0.134  (−0.330 to 0.062) | 0.101  (−0.003 to 0.204) |
| East Asia | −0.025  (−0.163 to 0.112) | −0.001  (−0.112 to 0.111) | −0.025  (−0.176 to 0.126) | −0.001  (−0.114 to 0.112) | −0.028  (−0.174 to 0.118) | −0.002  (−0.173 to 0.169) |
| Eastern Europe | −0.012  (−0.072 to 0.048) | −0.032  (−0.088 to 0.023) | −0.058  (−0.127 to 0.012) | −0.068  (−0.136 to 0.000) | 0.074  (−0.016 to 0.164) | 0.036  (−0.029 to 0.102) |
| Eastern Sub-Saharan Africa | 0.181  (−0.034 to 0.397) | 0.030  (−0.104 to 0.164) | −0.112  (−0.335 to 0.112) | −0.1  (−0.298 to 0.099) | −0.044  (−0.097 to 0.010) | −0.032  (−0.107 to 0.043) |
| High-income Asia Pacific | −0.096  (−0.193 to 0.001) | 0.012  (−0.021 to 0.045) | −0.076  (−0.147 to −0.005) | 0.012  (−0.025 to 0.048) | −0.114  (−0.223 to −0.005) | 0.005  (−0.025 to 0.036) |
| High−income North America | −0.010  (−0.021 to 0.001) | −0.006  (−0.013 to 0.001) | −0.003  (−0.006 to 0.000) | −0.002  (−0.005 to 0.000) | −0.003  (−0.007 to 0.000) | −0.004  (−0.009 to 0.000) |
| North Africa and Middle East | 0.042  (−0.079 to 0.163) | 0.012  (−0.098 to 0.121) | 0.014  (−0.124 to 0.152) | −0.047  (−0.164 to 0.070) | −0.002  (−0.098 to 0.094) | −0.021  (−0.112 to 0.070) |
| Oceania | 0.088  (−0.010 to 0.186) | 0.078  (0.003 to 0.154) | −0.067  (−0.135 to 0.001) | −0.074  (−0.138 to −0.010) | 0.015  (−0.064 to 0.094) | 0.018  (−0.052 to 0.088) |
| South Asia | 0.013  (−0.079 to 0.104) | −0.049  (−0.189 to 0.09) | 0.006  (−0.090 to 0.102) | −0.035  (−0.191 to 0.122) | 0.058  (−0.010 to 0.126) | 0.026  (−0.063 to 0.115) |
| Southeast Asia | 0.045  (−0.09 to 0.181) | −0.014  (−0.122 to 0.095) | −0.068  (−0.184 to 0.047) | −0.006  (−0.12 to 0.108) | −0.073  (−0.139 to −0.008) | −0.073  (−0.154 to 0.008) |
| Southern Latin America | 0.046  (−0.014 to 0.106) | −0.045  (−0.088 to −0.003) | −0.048  (−0.227 to 0.131) | 0.108  (0.014 to 0.202) | 0.015  (−0.005 to 0.036) | −0.005  (−0.016 to 0.007) |
| Southern Sub-Saharan Africa | 0.025  (−0.007 to 0.056) | 0.058  (−0.007 to 0.123) | −0.020  (−0.050 to 0.009) | −0.030  (−0.071 to 0.011) | −0.002  (−0.108 to 0.105) | 0.007  (−0.088 to 0.103) |
| Western Europe | −0.179  (−0.295 to −0.063) | −0.161  (−0.267 to −0.054) | −0.182  (−0.293 to −0.072) | −0.173  (−0.283 to −0.064) | −0.242  (−0.386 to −0.099) | −0.212  (−0.346 to −0.078) |
| Western Sub-Saharan Africa | 0.161  (−0.003 to 0.326) | 0.117  (0.005 to 0.229) | 0.112  (−0.102 to 0.327) | 0.093  (−0.096 to 0.282) | 0.041  (−0.098 to 0.181) | −0.016  (−0.129 to 0.097) |

GBD = global burden of disease; AMD = age-related macular degeneration
